# Structural insights into TSC complex assembly and GAP activity on Rheb

**DOI:** 10.1038/s41467-020-20522-4

**Published:** 2021-01-12

**Authors:** Huirong Yang, Zishuo Yu, Xizi Chen, Jiabei Li, Ningning Li, Jiaxuan Cheng, Ning Gao, Hai-Xin Yuan, Dan Ye, Kun-Liang Guan, Yanhui Xu

**Affiliations:** 1 Fudan University Shanghai Cancer Center, Institutes of Biomedical Sciences, State Key Laboratory of Genetic Engineering and Shanghai Key Laboratory of Medical Epigenetics, Shanghai Medical College of Fudan University, Shanghai, 200032 China; 2 The International Co-laboratory of Medical Epigenetics and Metabolism, Ministry of Science and Technology, China, Department of Systems Biology for Medicine, School of Basic Medical Sciences, Shanghai Medical College of Fudan University, Shanghai, 200032 China; 3 Human Phenome Institute, Collaborative Innovation Center of Genetics and Development, School of Life Sciences, Fudan University, Shanghai, 200433 China; 4 State Key Laboratory of Reproductive Regulation and Breeding of Grassland Livestock School of Life Sciences, Inner Mongolia University, Hohhot, 010070 China; 5 State Key Laboratory of Membrane Biology, Peking-Tsinghua Joint Center for Life Sciences, School of Life Sciences, Peking University, 100871 Beijing, China; 6 The Molecular and Cell Biology Research Lab, The Shanghai Key Laboratory of Medical Epigenetics, Institutes of Biomedical Sciences, Fudan University, Shanghai, 200032 China; 7 Department of Pharmacology and Moores Cancer Center, University of California San Diego, La Jolla, CA USA

**Keywords:** Tumour-suppressor proteins, Cell growth, Cell signalling, Cryoelectron microscopy

## Abstract

Tuberous sclerosis complex (TSC) integrates upstream stimuli and regulates cell growth by controlling the activity of mTORC1. TSC complex functions as a GTPase-activating protein (GAP) towards small GTPase Rheb and inhibits Rheb-mediated activation of mTORC1. Mutations in *TSC* genes cause tuberous sclerosis. In this study, the near-atomic resolution structure of human TSC complex reveals an arch-shaped architecture, with a 2:2:1 stoichiometry of TSC1, TSC2, and TBC1D7. This asymmetric complex consists of two interweaved TSC1 coiled-coil and one TBC1D7 that spans over the tail-to-tail TSC2 dimer. The two TSC2 GAP domains are symmetrically cradled within the core module formed by TSC2 dimerization domain and central coiled-coil of TSC1. Structural and biochemical analyses reveal TSC2 GAP-Rheb complimentary interactions and suggest a catalytic mechanism, by which an asparagine thumb (N1643) stabilizes γ-phosphate of GTP and accelerate GTP hydrolysis of Rheb. Our study reveals mechanisms of TSC complex assembly and GAP activity.

## Abstract (web summary)

Tuberous sclerosis complex (TSC) regulates cell growth by controlling the activity of mTORC1. The structure of human TSC complex reveals an arch-shaped, asymmetric architecture and a 2:2:1 stoichiometry of TSC1, TSC2, and TBC1D7 subunits and suggests a mechanism by which TSC2 accelerates GTP hydrolysis against a small GTPase Rheb.

## Introduction

The mechanistic target of rapamycin complex 1 (mTORC1) is a master regulator of cell growth by phosphorylating a variety of substrates, as exemplified by ribosomal S6 kinase 1 (S6K1) and eukaryote initiation factor 4E binding protein^1,2^. As a well-known tumor suppressor, the tuberous sclerosis complex (TSC) integrates cues of growth factors, energy status, and various stress to maintain Ras homolog enriched in brain (Rheb) in GDP-bound state, and therefore keeps mTORC1 in check to limit undesirable cell growth^2–5^. The TSC complex acts as a GTPase-activating protein (GAP) toward a small G-protein Rheb required for mTORC1 activation^6^. In GTP-bound state, Rheb directly binds to and activates mTORC1^7–11^.

The TSC complex consists of tuberous sclerosis complex 1 (TSC1), tuberous sclerosis complex 2 (TSC2), and an auxiliary subunit Tre2-Bub2-Cdc16-1 domain family member 7 (TBC1D7)^4,5^. Mutations of *TSC1* or *TSC2* genes cause tuberous sclerosis, an autosomal dominant genetic disease characterized by the development of histologically diverse hamartomas or benign tumors, including skin, brain, and kidneys^5,12^. TSC patients are frequently associated with severe neurological manifestations, including epilepsy, intellectual disability, and autism^5,13^. Although the functions of TSC complex have been extensively studied for decades, there are only a few structures of isolated domains, including TSC1 peptide bound to TBC1D7^3,14^, N-terminal domains of yeast TSC1^15^ and TSC2 (*Chaetomium thermophilum*)^16^, and recently reported TSC2 (*Chaetomium thermophilum*) GAP domain structure^17^. The lack of TSC complex structure has hampered understanding the mechanisms of complex assembly, GAP activity, and disease correlation.

Here we present the first cryo-EM structure of human TSC complex and elaborate on its characteristic assembly and GAP function. We propose a model of Rheb-bound TSC complex based on structural superimposition with GTP-bound Rheb^18^ and Rap1–Rap1GAP^19^ structures. Our structure also provides a framework for understanding the regulation of TSC complex function in mTORC1 pathway and its pathological significance.

## Results

### Structure determination

**Fig. 1:**
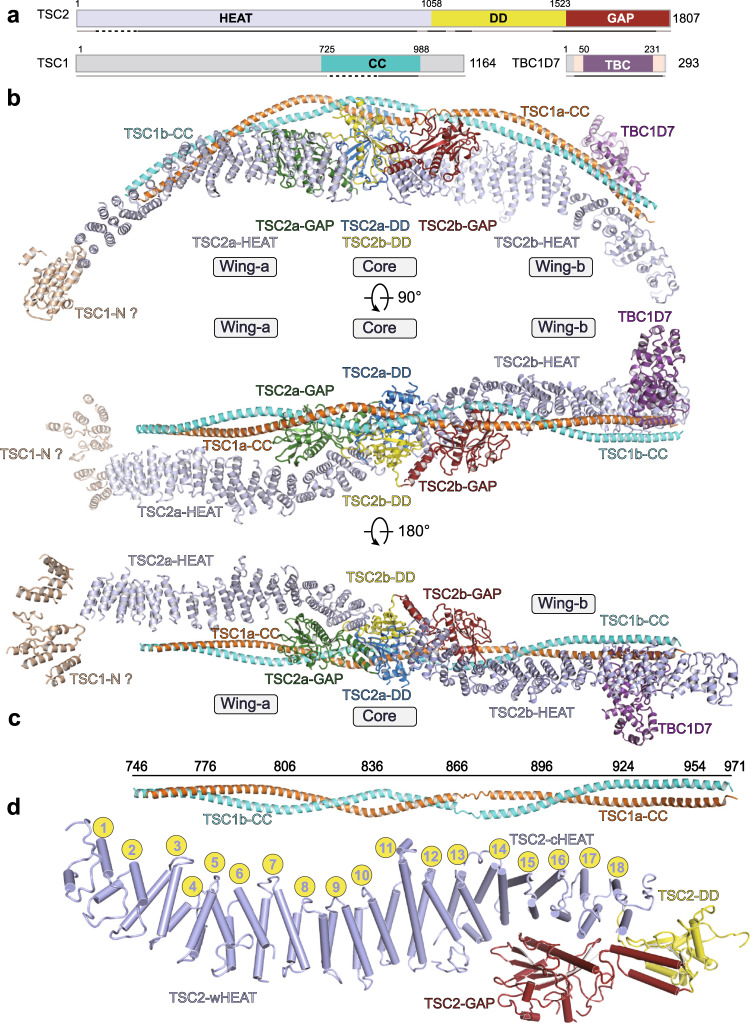
Overall structure of human TSC complex. **a** Color-coded domain structure of the three subunits with invisible regions colored in gray. Residues at domain boundaries are indicated with numbers. The same color scheme is used throughout all structure figures unless indicated elsewhere. HEAT: HEAT repeat domain, GAP: GTPase-activating domain, CC: coiled coil, DD: dimerization domain, TBC Tre2-Bub2-Cdc16 domain. Residues at domain boundaries are indicated. Unmodeled regions are indicated with gray lines under each protein. Solid black and dashed black lines below indicate regions that were modeled with residues and poly alanine, respectively. **b** Ribbon representation of TSC complex structure in three different views. The modules and subunits are labeled and indicated. **c** TSC1 coiled-coil dimer structure with residue positions indicated. **d** Cartoon model of one of TSC2 monomer, the HEAT repeats are indicated with numbered balls.

To obtain TSC complex structure, we overexpressed human TSC1, TSC2, and TBC1D7 in Expi293F cells and purified the complex to homogeneity (Supplementary Fig.1a). The purified TSC complex showed relatively weak in vitro GAP activity against Rheb, consistent with the known weak in vitro activity^8,19^ (Supplementary Fig.1b). The structure was determined by cryo-EM single particle reconstruction, and the cryo-EM map was refined to an overall resolution of 4.4 Å (Fig. 1b). The core and two wings were locally refined to 3.6, 3.9, and 4.1 Å resolution, respectively (Supplementary Figs. 2 and 3). The majority of structural model was unambiguously built ab initio aided by the structure of TSC1 fragment bound to TBC1D7^14^ and secondary structure analyses (Table 1 and Supplementary Movies 1–4). Residues of TSC1 (876–971), TSC2 (127–936, 1015–1082, 1182–1245, 1494–1732), and TBC1D7 (21–287) were traced and modeled with a TSC2a region (residues 127–315) and TSC1 (residues 746–875) being replaced by poly alanine due to the relatively weak cryo-EM density (Fig. 1a and Supplementary Fig. 4).

**Table 1:** Statistics of cryo-EM data collection, refinement, and validation statistics.

	TSC
Data collection and processing	
Magnification	×105,000
Voltage (kV)	300
Electron exposure (e^−^/Å^2^)	50
Defocus range (μm)	1.0–3.5
Pixel size (Å)	1.356
Symmetry imposed	C1
Initial particle images (No.)	1,528,982
Final particle images (No.)	131,022
Map resolution (Å)	
Consensus reconstruction	4.4
Focus wing-a reconstruction	4.1
Focus core reconstruction	3.6
Focus wing-b reconstruction	3.9
FSC threshold	0.143
Map resolution range (Å)	
Consensus reconstruction	4.0–20.0
Focus wing-a reconstruction	4.0–20.0
Focus core reconstruction	3.0–10.0
Focus wing-b reconstruction	3.0–10.0
Refinement	
Initial model used (PDB code)	5EJC
Model resolution (Å)	4.5
FSC threshold	0.5
Model resolution range (Å)	4.0–4.5
Map sharpening B factor (Å^2^)	−129.92
Model composition	
Non-hydrogen atoms	23,934
Protein residues	3089
Ligands	0
B factors (Å^2^)	
Protein	141.96
Ligand	–
RMS deviations	
Bond lengths (Å)	0.004
Bond angles (°)	0.673
Validation	
MolProbity score	2.42
Clashscore	24.52
Poor rotamers (%)	0.12
Ramachandran plot	
Favored (%)	90.30
Allowed (%)	9.70
Disallowed (%)	0.00

### Overall structure of human TSC complex

The TSC complex consists of a central core and two wings (termed wing-a and wing-b) and the overall structure exhibits an elongated arch-shaped fold with approximate dimensions of ~390 × 133 × 88 Å^3^ (Fig. 1b). TSC1, TSC2, and TBC1D7 assemble the TSC complex with a 2:2:1 stoichiometry, generating an asymmetric modular organization. The overall architecture of TSC1–TSC2 and TSC1–TBC1D7 interactions together allows one TBC1D7 to be assembled into TSC complex and this subunit stoichiometry is consistent with previous structural and biochemical analyses^3,14^. Although isolated domains adopt similar folds, the two TSC1 (termed TSC1a/1b) and two TSC2 (TSC2a/2b) reveal distinct conformations, respectively (Supplementary Fig. 5). The two TSC2 molecules form a pseudo-symmetric dimer through tail-to-tail interactions. The coiled-coil domains (CCs, residues 746–971) of TSC1a and TSC1b interwind in parallel and form an extended two-helix bundle with the dimer interface being as much as 2386 Å^2^ (Fig. 1c and Supplementary Fig. 6a–d). The TSC1 dimer makes multiple contacts with TSC2 dimer and stabilizes the overall conformation of the complex. This parallel dimerization of TSC1 leads to an asymmetric formation of TSC1–TSC2 tetramer and recruitment of a single TBC1D7 molecule, generating a unique and characteristic modular organization (Fig. 1b and Supplementary Fig. 5f, g).

Each TSC2 consists of a HEAT repeat domain (HEAT), a dimerization domain (DD), followed by a C-terminal GAP catalytic domain (GAP) (Fig. 1a, b, d). The N-terminal 12 HEAT repeats (wing HEAT, wHEAT) flank out of the central core and are stabilized by TSC1. The following six HEAT repeats (core HEAT, cHEAT) associate with and are stabilized by the central GAP and DD domains. The cHEAT–DD–GAP of the two TSC2 molecules adopt pseudo-symmetric fold, whereas the two wHEAT domains adopt distinct conformations due to differently associated TSC1 (Supplementary Fig. 5). TSC2a and TSC2b bind the N-terminal (N-CC) and C-terminal (C-CC) halves of the TSC1 CC dimer, respectively (Fig. 1b).

The TBC1D7 associates with and stabilizes the C-terminal helices (residues 937–971) of TSC1a/1b but has no direct contact with TSC2. The TBC1D7–TSC1a/1b module adopts a similar fold to the human TSC1–TBC1D7 crystal structure^14^ (Supplementary Fig. 6e), and is positioned far away from the central core, consistent with its auxiliary role in TSC complex assembly and function^4^. The observation agrees with the immunoprecipitation results showing that TBC1D7 binds TSC1 but not TSC2 (Supplementary Fig. 6f).

### TSC1 structure and its interaction with TSC2

The two CC domains of TSC1a and TSC1b are paired in parallel and form a two-turn left-handed supercoil (Fig. 1c). The pairwise CC involves extensive intermolecular contacts. The TSC1 homodimer interface is enriched in nonpolar residues, which make extensive hydrophobic contacts to support a stable TSC1 dimerization and its scaffolding function (Supplementary Fig. 6a–d).

**Fig. 2:**
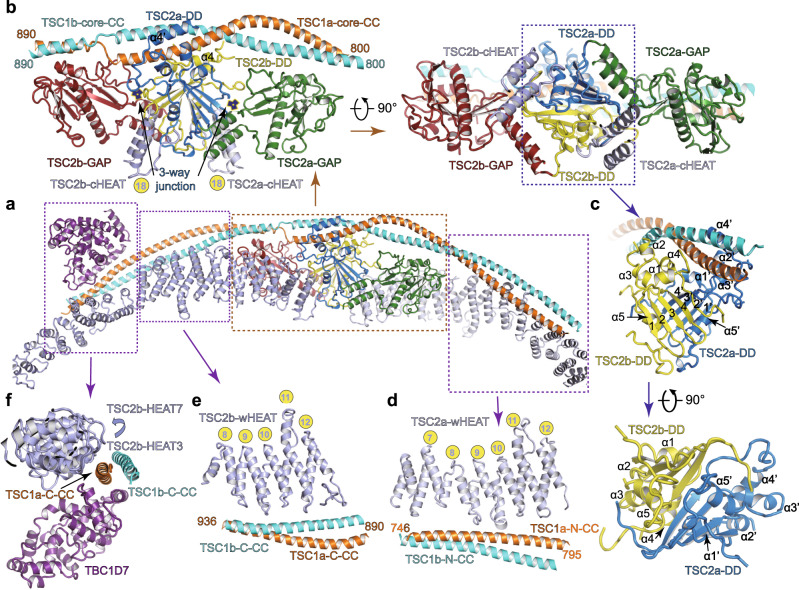
Intermolecular interfaces of TSC complex. **a** Overall structure of TSC complex shown in a view different from that in Fig. 1b, intermolecular contacts shown in (**b**–**f**) are highlighted with dashed boxes. **b** Close-up view of the intermodular interactions in the central core module. Close-up view of the intermodular interactions in the dimerization domain (**c**), wing-a (**d**), wing-b (**e**), and TSC1 C-CC with TBC1D7 (**f**). Critical elements are indicated. In (**c**), α1–α5 and α1′–α5′ represent α helices of TSC2b and TSC2a, respectively. The numbers (1–4 and 1′–4′) represent the numbered β strands.

The TSC1 CC dimer adopts an arch-shaped architecture and packs against the ridge of TSC2 dimer (Figs. 1b and 2a). The CC dimer makes four major contacts with TSC2. (1) The central region of TSC1a (residues 800–890) sits on a “saddle” formed by TSC2 DD dimer (Fig. 2b, c). (2) The TSC1a-N-CC (residues 746–795) packs against the ridge of repeats HEAT7–HEAT12 of TSC2a (Fig. 2d). (3) The C-CC (residues 890–936) dimer of TSC1 packs against the ridge of the repeats HEAT8–HEAT12 of TSC2b (Fig. 2e). (4) The TSC1a–C-CC (residues 937–971) packs against the ridge of repeats HEAT3–HEAT7 of TSC2b (Fig. 2f), confirmed the known interaction between the N-terminus of TSC2 (residues 1–418) and TSC1^16,20,21^. Consistent with the asymmetric complex formation, TSC1a plays a major role in binding TSC2 dimer. In support of above structural observation, our immunoprecipitation assay shows that full-length TSC1 binds N-HEAT and weakly associates with DD–GAP but exhibits undetectable interaction with DD or GAP domain of TSC2 (Supplementary Fig. 6i).

Another asymmetric feature of the TSC complex exists around the end of wing-a module. The cryo-EM map reveals repetitive helical region covering the N-terminal HEAT repeats of TSC2a but not TSC2b. This region is likely derived from the predicted N-terminal HEAT repeats domain of TSC1 (Fig. 1b and Supplementary Fig. 4b)^15^. Other TSC1 regions were invisible in our cryo-EM map due to flexibility. In our immunoprecipitation assay, the full-length and CC of TSC1 shows comparable binding to TSC2 (Supplementary Fig. 6g), consistent with the maintenance of TSC1–TSC2 upon deletion of TSC1 several N-terminal fragments^22^.

### Domain organization and dimerization of TSC2

The TSC2 monomer adopts a seahorse-shaped conformation, in which the wHEAT (HEAT1–HEAT12) and cHEAT (HEAT13–HEAT18) together adopt a right-handed super helical fold (Fig. 1d). In the two TSC2 molecules, the isolated cHEAT and wHEAT domains adopt almost identical conformations, respectively. However, the whole HEAT domain of TSC2b tends to be more extended than that of TSC2a (Supplementary Fig. 5e–g). The two HEAT domains bind the TSC1 dimer in a distinct manner, suggesting that different features of N- and C-terminal portions of TSC1 CC dimer lead to distinct conformation of the two TSC2 molecules (Supplementary Fig. 5f).

The cHEAT–DD–GAP parts of two TSC2 molecules form an almost symmetrical core of the complex and the dimerization are mediated by two stably associated DD domains (Fig. 2a–c and Supplementary Fig. 5c–e). Each DD domain consists of a four-stranded antiparallel β-sheet (Dβ1–Dβ4) and five flanking α-helices (Dα1–Dα5) (Fig. 2c). The two β-sheets together form a saddle-shaped eight-stranded β-sheet. The Dα5 helix (residues 1472–1483) packs against the concave surface of the saddle and binds the other TSC2 molecule on HEAT18 and the following loop (residues 1024–1038) (Fig. 2a and Supplementary Fig. 7). The loop preceding helix Dα4 inserts into a hydrophobic pocket of the other TSC2 molecule located in a three-way junction formed by the cHEAT, DD, and GAP domains (Fig. 2a). The TSC2 dimerization is further supported by DD helices of the two TSC2 molecules, which sandwich the TSC1a CC domain. Around this region, the TSC2 dimer interface (~2805 Å^2^) is larger than TSC1–TSC2 interface (~1761 Å^2^), suggesting that TSC2 may form a homodimer independent of TSC1 and the TSC2 dimer is required for generating a stable TSC1–TSC2 tetramer^23^.

### The TSC2 GAP structure and its positioning in TSC complex

**Fig. 3:**
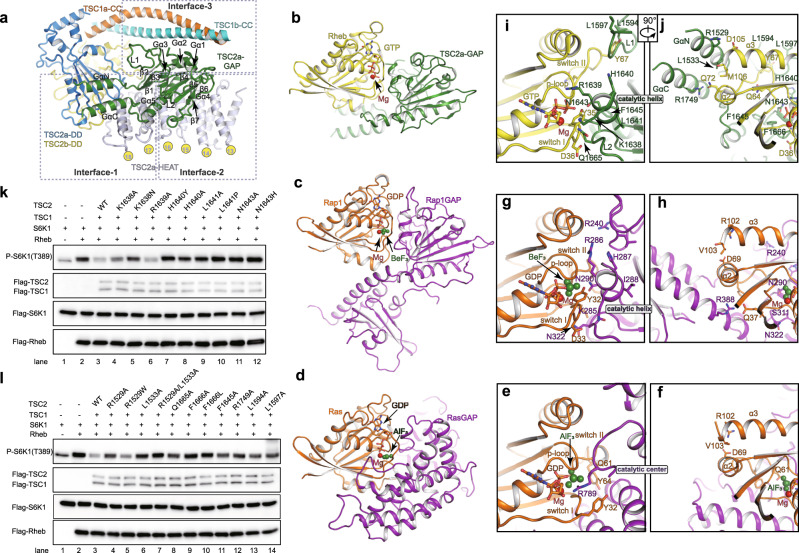
TSC2 GAP catalytic mechanism and putative GAP–Rheb binding. **a** Close-up view of TSC2a GAP domain and its positioning. Three inter-domain contacts are highlighted with dashed boxes. Structural comparison of Rheb–TSC2 GAP (**b**), Rap1–Rap1GAP (PDB:3BRW) (**c**), and Ras–RasGAP (PDB:1WQ1) (**d**). The structures are shown in a similar view. TSC2 GAP and Rheb are shown in green and yellow, respectively. Rap1 and Ras are colored in orange and Rap1GAP and RasGAP are colored in magenta, respectively. In (**b**), the GTP-bound Rheb (PDB:1XTS) and TSC2 GAP domain were, respectively, superimposed to Rap1 and Rap1GAP in Rap1–Rap1GAP structure. Two different close-up views of the catalytic centers of RasGAP (**e**, **f**), Rap1GAP (**g**, **h**), and TSC2 (**i**, **j**). The structures are derived from (**b**–**d**). Magnesium cations are shown as red balls. The beryllium trifluoride (BeF_3_) and aluminum trifluoride (AlF_3_) are shown as green balls. The GDP–BeF_3_ and GDP–AlF_3_ are the mimetic ATP in ground and transition states, respectively. Residues involved in binding and catalysis are shown in sticks. **k**, **l** Cell-base GAP activity assays of wild-type TSC2 and TSC2 mutants. The HEK293A cells were transfected with (+) or without (−) the indicated plasmids in the upper of the panel. The activities were detected by western blotting with antibody against phosphorylated-S6K (T389). The effects of residues involved in catalysis (**k**) and Rheb binding (**l**) were tested. Source data are provided as a Source Data file for uncropped blots.

The two GAP catalytic domains are symmetrically cradled within the central core module and each GAP adopts a characteristic mixed α/β fold (Figs. 1b and 3a). A central seven-stranded β-sheet is stabilized by a long α helix (Gα5) from the concave side. The helix Gα3 (catalytic helix) and two loops (L1 and L2) pack against the convex surface of the β-sheet. Three intermodular contacts involve positioning of each GAP domain (Fig. 3a and Supplementary Fig. 8). (1) Two parallel α helices (GαN and GαC) form a GAP extension, which protrudes out of the catalytic core and binds the edge of the β-sheet of the DD domain and repeats HEAT17–HEAT18 (Supplementary Fig. 8b). (2) The helix Gα5, strand Gβ7, and its preceding loop, together pack against the concave surface of repeats HEAT13–HEAT16 (Supplementary Fig. 8c). (3) The helix Gα1 (TSC2b) or Gα2 (TSC2a) and loop L1 bind TSC1 CC and the binding pattern is slightly different in two GAP domains. The GAP of TSC2a binds single CC (residues 800–830) of TSC1b whereas the GAP of TSC2b binds two CC strands (residues 860–895) of TSC1a/1b (Supplementary Fig. 8d). The lack of TSC1 largely decreased the GAP activity, indicating that TSC1 is required for assembly of fully active GAP domains in TSC complex (Supplementary Fig. 6h). Consistent with above cellular experiments, TSC complex possessed relatively weak GAP activity in the in vitro assay while the isolated TSC2 GAP domain showed undetectable activity (Supplementary Fig. 6j). The two GAP catalytic core adopt almost identical conformations and their catalytic pockets both open outwards, suggesting a similar manner of substrate recognition and catalysis (Fig. 2a and Supplementary Fig. 5d, e).

### Catalytic mechanism of TSC2 GAP

The TSC2 GAP domain is highly conserved from yeast to human and shares considerable sequence homology to Rap1GAP (Supplementary Fig. 9a), suggesting that TSC2-stimulated GTP hydrolysis of Rheb follows the same mechanism as in Rap–Rap1GAP system^8,19,21^. To investigate the mechanism of TSC2-stimulated GTP hydrolysis of Rheb, we superimposed our TSC2 GAP structure and Rheb–GTP (PDB: 1XTS)^18^ with Rap1–Rap1GAP structure (PDB: 3BRW)^19^ and the classical small G-protein Ras–RasGAP (PDB: 1WQ1)^24^ (Fig. 3b–d). Structural comparison confirms the predicted structural similarity between the GAP domains of TSC2 and Rap1GAP and reveals distinct fold of the associated domains, which may provide substrate specificity (Fig. 3b, c).

Previous structural and biochemical studies of small GTPases and their GAPs have proposed a generally conserved activation mechanism^25,26^. All the GAP domains provide positively charged residues, neutralize negative charges generated during phosphoryl transfer reactions, and thus accelerate GTP hydrolysis^25,26^. Ras–RasGAP represents the prototypic small GTPase–GAP system, in which a *trans*-arginine finger (R789 in RasGAP) and a *cis*-glutamine (Q61 in Ras) are critical for catalysis through stabilizing the γ-phosphate in the transition state^24,27^ (Fig. 3e, f). The arginine finger is shared by GAPs of some other Ras superfamily members^26^. As a representative exception, Rap1–Rap1GAP lacks the arginine finger, but instead, has an asparagine thumb (N290 in Rap1GAP), which stabilizes γ-phosphate and is essential for GAP activity^19,28^ (Fig. 3g, h).

To analyze the structure of TSC2 GAP, we generated a model of Rheb-bound TSC complex through superimposition of TSC2 GAP and GTP-bound Rheb^18^ to Rap1–Rap1GAP^19^ complex structure (Fig. 3b, c). Structural superimposition suggests that Rheb binds TSC2 GAP in a manner similar to that in Rap1–Rap1GAP. The catalytic helix (Gα3, K^1638^RHLGN^1643^) of TSC2 faces toward the catalytic cavity formed by the switch I, switch II, and P-loop of the superimposed Rheb (Fig. 3i, j). The switch I is conserved among the small G proteins (Supplementary Fig. 9b). Residue N1643 of TSC2 is similarly positioned to N290 of Rap1GAP, suggesting a shared asparagine thumb of the two GAP domains. As an equivalent of residue Y32 of Rap1 and Y32 in Ras, residue Y35 of Rheb is positioned close to N1643 and may facilitate GTP hydrolysis. The TSC2 catalytic helix is positioned similar to that of Rap1GAP in Rap1–Rap1GAP^19^ complex structure, suggesting a similar molecular environment in stabilizing catalytic helix in the two GAP domains (Fig. 3g, i and Supplementary Fig. 10c). It has been known that Rap1GAP forms a dimer through a DD. Although the central β-sheet of the DD domains of Rap1GAP and TSC2 could be aligned well, the DD–GAP dimers adopt distinct modular organization (Supplementary Fig. 10d).

Other residues of the catalytic helix may support the catalytic helix conformation (Fig. 3i, j). Residues K1638 and R1639 (equivalent to K285 and R286 of Rap1GAP) face toward the putative Rheb and their mutations may affect substrate binding. Residue L1641 (equivalent to I288 of Rap1GAP) faces toward the core of TSC2 GAP. H1640 (equivalent to H287 of Rap1GAP) is in proximity to and binds residue R15 of Rheb, which is consistent with the model presented in the recent report^17^. The conformational stability of the equivalent catalytic helix of RasGAP is essential for its activity^6,24^, suggesting that these catalytic helix residues may also be required for GAP activity (Fig. 3e, g, i).

We performed cell-based assay to investigate the GAP activity of TSC complex by detecting the phosphorylation of S6K1 at T389, which is well accepted to represent the level of Rheb in GTP-bound form and TSC2 GAP activity^8,29^ (Fig. 3k). The co-transfection of TSC1 and TSC2 largely decreased the level of phosphorylated-S6K1, indicating a robust GAP activity in cells (Fig. 3k, lanes 1–3). Alanine substitutions of K1638 or R1639 and tuberous sclerosis-associated mutation of K1638 showed weak to moderate defect on GAP activity (Fig. 3k, lanes 4–6). K1638N mutation may hinder substrate binding due to steric hindrance. Alanine substitutions or tuberous sclerosis-associated mutations of H1640, L1641, or N1643 on TSC2, largely impaired the GAP activity (Fig. 3k, lanes 7–12), to a level comparable to that of lacking TSC complex (Fig. 3k, lane 2).

The result is consistent with structural observation and supports the notion that TSC2 uses the asparagine thumb (N1643) to accelerate GTP hydrolysis of Rheb and residues K1638, H1640, and L1641 of the catalytic helix function in supporting the conformation of the asparagine thumb. Our structural and biochemical studies show that L1641 plays an important role in TSC2 GAP activity and confirmed the significance of residues K1638, H1640, N1643, which have been shown to be critical for TSC2 GAP activity in previous studies^10,30,31^.

### The Rheb recognition by TSC2 GAP domain

Structural model of Rheb-bound TSC complex (generated from structure superimposition) suggests that Rheb is well accommodated by the TSC2 GAP domain and has no clash with other domains. This putative Rheb–TSC2 binding pattern differs from that of Rap1–Rap1GAP and Ras–RasGAP due to characteristic features of TSC2 GAP, which may confer specificity toward Rheb. Besides the catalytic helix, the loops L1 and L2 are, respectively, positioned close to switch II and switch I of the superimposed Rheb, possibly generating two putative TSC2–Rheb contacts (Fig. 3b–j and Supplementary Fig. 8e). Previous study reported that L1594 and F1666 mutations decreased TSC2 GAP activity^30^. Our GAP assay shows that Alanine substitutions or disease-associated mutations of L1 (L1594, L1597), L2 (Q1665, F1666), and F1645 impaired its GAP activity, confirming their supportive roles in substrate recognition and/or catalysis (Fig. 3l, lanes 13–14 and 8–11).

The structure reveals a characteristic helix pair formed by GαN (residues 1525–1536) and GαC (residues 1739–1754) of TSC2. The helix pair is positioned near the helices α2 (residue Q72) and α3 (residues D105 and M106) of the putatively bound Rheb and likely supports TSC2–Rheb interactions (Fig. 3f, h, j). Mutations R1529A, L1533A, and double mutation R1529A/L1533A on GαN and R1749A on GαC led to moderate to severe decrease in TSC2 GAP activity, suggesting their critical roles in supporting TSC2–Rheb contacts (Fig. 3l, lanes 4–7 and 12). Previous study also showed that R1749Q mutation decreased the GAP activity to some extent^21^.

During our paper preparation, Hansmann et al.^17^ reported a crystal structure of isolated *C. thermophilum* TSC2 GAP domain. The structure reveals a similar fold to that of TSC2 GAP domain, consistent with relatively high sequence similarity (Supplementary Fig. 9a, b). The proposed mechanism of GAP activity on Rheb is also consistent with our independent studies. However, the isolated *C. thermophilum* GAP forms a monomer and lacks the helix pair extension, likely because the DD domain and helices GαN/GαC have been truncated during protein preparation (Supplementary Fig. 10a, b). Recently, Ramlaul et al.^32^ reported the architecture of TSC complex at a relatively lower resolution in BioRxiv, which confirmed generally similar overall fold of TSC complex in our study.

## Discussion

**Fig. 4:**
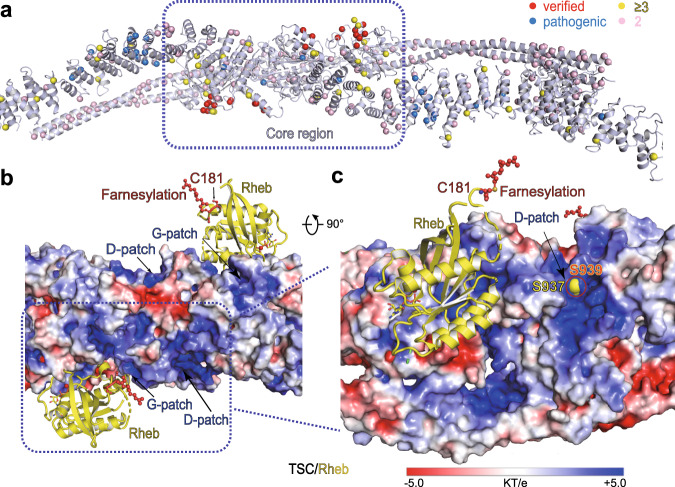
TSC complex surface feature and functional implications. **a** The cartoon structure of TSC complex are shown with cancer-associated mutations highlighted with colored balls. The information of patient-derived mutations was obtained from the Catalogue of Somatic Mutations in Cancer (COSMIC)^33^. Critical residues for GAP activity that were verified in our study are shown as red balls, pathogenic mutations are shown as blue balls, and the mutations identified in patients ≥3 and 2 times in COSMIC database are shown as yellow and pink balls, respectively. **b**, **c** Electrostatic potential surface of TSC complex is shown in two views. Two putative Rheb molecules (yellow) are shown in cartoon with farnesylation at C181 (red) indicated (**b**). The traced residue S937 is shown in yellow balls. The positively charged patches around dimerization domain (D-patch) and GAP domain (G-patch) are shown in a close-up view.

Mutations of *TSC* genes have been frequently observed in tuberous sclerosis and cancers and missense mutations occurred throughout the protein sequences^33^ (Fig. 4a and Supplementary Fig. 4). Notably, most of cancer-derived mutations and functionally important residues in *TSC2* are enriched on the central core module, supporting the pathological significance of TSC complex in these diseases. Furthermore, the identified pathogenic mutations in the wing modules are predominantly enriched on the ridges of HEAT domains of TSC2, consistent with their roles in mediating TSC1–TSC2 interactions and TSC complex conformational stability^21,31,34–38^.

The surface electrostatic calculations of TSC2 structure reveals four predominant positively charged patches around the DD (D-patch) and GAP (G-patch) domains. The four patches are located on the bottom surface of the central core and close to the putative Rheb-binding pockets of the two GAP domains, suggesting a regulatory role related to its GAP function (Fig. 4b, c). It is tempting to speculate that these positively charged patches may involve charge–charge interactions and associate with negatively charged phosphorylated residues and/or lipids.

It is well documented that residue S939 of TSC2 is phosphorylated by the AKT kinase and the phosphorylated TSC2 is translocated from lysosomal membrane to cytosol via binding of 14-3-3 protein, and therefore inhibits GAP activity on Rheb and activates mTORC1 activity^39,40^. Although residue S939 was invisible due to the lack of corresponding cryo-EM density, its nearest modeled residue S937 is located around the D-patch (Fig. 4c), which may recruit and stabilize the phosphorylated S939.

It is well known that inactivation of mTORC1 requires TSC2 lysosomal localization^41^ and TSC2 is recruited to lysosome membrane through nonexclusive pathways, such as binding C181 farnesylated Rheb^42^, Rag GTPases^43^, and polycystin-1^44^. Structural superimposition indicates that the farnesylated Rheb has no clash with the positive patches of TSC2, supporting its co-localization with TSC complex on lysosomal surface (Fig. 4b and Supplementary Fig. 8e). Lipid phosphorylation has been known to regulate membrane localization of proteins^45^. TSC complex may bind phosphorylated lipid on lysosome membrane via its positive patches through charge–charge interactions, providing an alternative approach for its lysosomal localization.

## Methods

### Reagents

Flag-M2 affinity agarose gel was from Raygene; Mono Q and Superose 6 were from GE Healthcare; polyethylenimine (PEI) was from Polysciences (23966); HEK293A and Expi293F cells were from Invitrogen Inc.; and culture medium was from Sino Biological Inc. Antibodies against phosphorylated-S6K (Thr 389) were from Cell Signaling Technology; Flag-HRP (A8592) was from Sigma; and Horseradish peroxidase-labeled anti-mouse and anti-rabbit secondary antibodies were from AbMart.

### Protein expression and purification

The ORFs of human TSC1, TSC2, and TBC1D7 were sub-cloned into three modified pCAG vectors. The three plasmids were co-transfected to suspension Expi293F cells using PEI. After culture at 37 °C, 5% CO2 for 3 days, cells were collected and lysed in 50 mM HEPES (pH 7.4), 300 mM NaCl, 0.2% CHAPS, 5 mM MgCL_2_, 5 mM ATP, 10 mM NaF, and 3 mM DTT at 4 °C for 30 min, and the insoluble fraction was removed by centrifugation at 38,000 × *g* for 30 min. Supernatants were incubated with Flag-M2 monoclonal antibody-agarose for 4 h and washed extensively. The fusion proteins (Flag-tagged TSC1, Myc-tagged TSC2, and Myc-tagged TBC1D7) were digested using PreScission protease overnight and the eluted proteins were further purified using ion exchange and gel filtration chromatography. The peak fractions were pooled for gradient fixation (Grafix)^46^. The gradient was generated from a 10% glycerol light solution (10% (v/v) glycerol, 300 mM NaCl, 25 mM HEPES (pH 7.4), 1 mM TCEP), and a 30% glycerol heavy solution (30% (v/v) glycerol, 300 mM NaCl, 25 mM HEPES (pH 7.4), 1 mM TCEP, and 0.1% (v/v) glutaraldehyde). Centrifugation was performed at 247,605 × *g* in a SW41Ti swinging bucket rotor for 18 h at 4 °C using Beckman L-100XP. Subsequently, peak fractions were collected and quenched with 100 mM Tris-HCl (pH 8.0). The cross-linked TSC complex was concentrated and dialyzed to 0.5 mg/ml for Cryo-EM grids.

### Sample preparation

For negative staining EM grids preparation, 5 µL of TSC complex sample was applied onto glow-discharged copper grids supported by a continuous thin layer of carbon film for 60 s before negatively stained by 2% (w/v) uranyl formate solution at room temperature. The grids were prepared in the Ar/O_2_ mixture for 15 s using a Gatan 950 Solarus plasma cleaning system with a power of 35 W. The negatively stained grids were loaded onto a Thermo Fisher Scientific Talos L120C microscope equipped with a Ceta CCD camera and operating at 120 kV at a nominal magnification of ×92,000, corresponding to a pixel size of 1.58 Å on the specimen.

For cryo-EM grids preparation, 4 μL of the sample at a concentration of ~0.5 mg/mL TSC complex was applied to freshly glow-discharged Quantifoil R1.2/1.3 holey carbon grids. After incubation of 5 s at a temperature of 4 °C and a humidity of 100%, the grids were blotted for 4–6 s in a Thermo Fisher Scientific Vitrobot Mark IV and plunge-frozen in liquid ethane at liquid nitrogen temperature. The grids were prepared in the H_2_/O_2_ mixture for 60 s using a Gatan 950 Solarus plasma cleaning system with a power of 5 W. The ø 55/20 mm blotting paper is made by TED PELLA used for plunge freezing.

### Data collection

The cryo-EM grids of TSC complex were loaded onto a Thermo Fisher Scientific Titan Krios transmission electron microscope equipped with a Gatan GIF Quantum energy filter (slit width 20 eV) and operating at 300 kV for data collection. All the cryo-EM images were automatically recorded by a post-GIF Gatan K2 Summit direct electron detector in the super-resolution counting mode using Serial-EM^47^ with a nominal magnification of ×105,000 in the EFTEM mode, which yielded a super-resolution pixel size of 0.678 Å on the image plane, and with a defocus ranged from 1.0 to 3.5 μm. Each micrograph stack was dose-fractionated to 32 frames with a total electron dose of ~50 e^−^/Å^2^ and a total exposure time of 11.49 s. For the first data set of TSC complex sample, 3316 micrographs from a total of 3605 micrographs were selected for further processing. As for the second data set of TSC complex sample, 1381 micrographs from a total of 1546 micrographs were selected for further processing.

### Image processing

For cryo-EM data, drift- and beam-induced motion correction was applied on the super-resolution movie stacks using MotionCor2^48^ and binned twofold to a calibrated pixel size of 1.356 Å/pix. The defocus values were estimated by Gctf^49^ from summed images without dose weighting. Other procedures of cryo-EM data processing were performed within RELION v3.0^50,51^ using the dose-weighted micrographs.

For the first data sets of the TSC complex, a subset of ~10,000 particles was picked by Gautomatch (Zhang unpublished) without reference and subjected to reference-free 2D classification. Some of the resulting 2D class averages were low-pass filtered to 15 Å and used as references for automatic particle picking of the whole data sets in RELION resulting in an initial set of 1,073,891 particles for reference-free 2D classification. In all, 510,614 particles were selected from good 2D classes for the initial 3D classification, using a 60 Å low-pass filtered initial model from our previous cryo-EM reconstruction. After several rounds of 2D and 3D classification, 152,396 particles were 3D auto-refined and post-processed, yielding a reconstruction of TSC complex at 5.11 Å resolution. Also, for the second data set of the TSC complex, a subset of ~10,000 particles was picked by Gautomatch (Zhang unpublished) without reference and subjected to reference-free 2D classification. Some of the resulting 2D class averages were low-pass filtered to 20 Å and used as references for automatic particle picking of the whole data sets in RELION resulting in an initial set of 455,091 particles for reference-free 2D classification. Overall, 244,896 particles were selected from good 2D classes for the initial 3D classification, using a 60 Å low-pass filtered initial model from our previous cryo-EM reconstruction. After several rounds of 2D and 3D classification, 71,265 particles were 3D auto-refined and post-processed, yielding a reconstruction of TSC complex at 5.22 Å resolution. According to these reconstructions, TSC^dataset1^ and TSC^dataset2^ are the same sample. Thus, two data sets were merged to improve the map quality. After several rounds of 2D and 3D classification, 131,022 particles were 3D auto-refined and post-processed, yielding a reconstruction at 4.4 Å resolution. We used a local mask 3D refinement for the wing-a, core, and wing-b region, 131,022 particles were local refined and post-processed, yielding a 4.1 Å reconstruction of TSC complex wing-a region, a 3.6 Å reconstruction of TSC complex core region, and a 3.9 Å reconstruction of TSC complex wing-b region, respectively.

All reported resolutions are based on the gold-standard Fourier shell correlation (FSC) = 0.143 criterion. The GSFSC curves were corrected for the effects of a soft mask with high-resolution noise substitution. All cryo-EM maps were sharpened by applying a negative B-factor estimated during post-processing in RELION. All the visualization and evaluation of the 3D volume map were performed within UCSF Chimera or UCSF ChimeraX^52^, and the local resolution variations were calculated using RELION^50^.

### Model building and structure refinement

The cryo-EM maps of the TSC complex wing-a region complex at 4.1 Å resolution, the TSC complex core region complex at 3.6 Å resolution, and the TSC complex wing-b region complex at 3.9 Å resolution were used for model fitting. The structure of TSC1–TBC1D7 (PDB: 5EJC) was used as initial structural template, which was docked into the cryo-EM maps by rigid-body fitting using UCSF Chimera^52^. The structural models were further manually built de novo in COOT^53^ and refined in real space using Phenix^54^ with secondary structure and geometry restraints using the cryo-EM map. Overfitting of the model was monitored by refining the model in one of the two half maps from the gold-standard refinement approach and testing the refined model against the other map^55^. Statistics of the map reconstruction and model refinement can be found in Table 1. The final models were evaluated using MolProbity^56^. Map and model representations in the figures and movies were prepared by PyMOL (http://www.pymol.org*)*, UCSF Chimera, or UCSF ChimeraX^57^.

### In vitro GAP assay

GTPase-activating activity was determined with a calorimetric assay^58^ measuring the formation of inorganic phosphate. The purified TSC complex or TSC2 GAP domain was incubated with Rheb (3 μM) in a buffer containing 25 mM Hepes (pH 7.4), 200 mM NaCL, 1 mM GTP in 50 μL reaction mixtures and incubated at 37° for 3 h. Reactions were terminated by the addition of 100 μL of malachite green/acid molybdate solution. After 20 min of color development, OD620 was determined.

### In vivo GAP assay

The HEK293A cells were transfected with Flag-S6K1, Flag-Rheb, Flag-TSC1, Myc-TBC1D7, Flag-TSC2 WT, and mutants using PEI. After 48 h, the cells were collected and lysed for 30 min. The supernatant was collected by centrifuge and boiled with SDS loading buffer. The sample was conducted for western blotting. The primary antibody was incubated overnight and washed three times with TBST, and incubated with secondary antibody for 1 h. After extensive rinsing with TBST for three times, ECL was detected.

### Reporting summary

Further information on research design is available in the Nature Research Reporting Summary linked to this article.

## Supplementary information

Supplementary Information

Supplementary Movie 1

Supplementary Movie 2

Supplementary Movie 3

Supplementary Movie 4

Reporting Summary

Description of Additional Supplementary Files

## Data Availability

The electron density map and corresponding atomic coordinates have been deposited in the Protein Data Bank (http://www.rcsb.org/pdb) under accession code: 7DL2 and in EMDB under accession codes: EMD-30708, EMD-30709, EMD-30710, and EMD-30711. Source data are provided with this paper.

## Source data

Source Data
