# Supplementary material for: Structural insights into TSC complex assembly and GAP activity on Rheb
Source: Nat Commun. 2021 Jan 12;12:339. doi: 10.1038/s41467-020-20522-4 (PMC7804450; doi:10.1038/s41467-020-20522-4)
Supplement: Supplementary file 1 — Supplementary Information [file 41467_2020_20522_MOESM1_ESM.pdf]

Supplementary Information For

Structural insights into TSC complex assembly and GAP activity on Rheb

Huirong Yang[1,2,3,4 #], Zishuo Yu[1#], Xizi Chen[1#], Jiabei Li[1], Ningning Li[5], Jiaxuan Cheng[5], Ning Gao[5], Hai-Xin Yuan[6], Dan Ye[6], Kun-liang Guan[7], and Yanhui Xu[1,2,3,4]*

[1]Fudan University Shanghai Cancer Center, Institutes of Biomedical Sciences, State Key Laboratory of Genetic Engineering and Shanghai Key Laboratory of Medical Epigenetics, Shanghai Medical College of Fudan University, Shanghai 200032, China

[2]The International Co-laboratory of Medical Epigenetics and Metabolism, Ministry of Science and Technology, China, Department of Systems Biology for Medicine, School of Basic Medical Sciences, Shanghai Medical College of Fudan University, Shanghai 200032, China

[3]Human Phenome Institute, Collaborative Innovation Center of Genetics and Development, School of Life Sciences, Fudan University, Shanghai 200433, China

[4]State Key Laboratory of Reproductive Regulation and Breeding of Grassland Livestock School of Life Sciences, Inner Mongolia University, Hohhot, 010070, China

[5]State Key Laboratory of Membrane Biology, Peking-Tsinghua Joint Center for Life Sciences, School of Life Sciences, Peking University, Beijing 100871, China

[6]The Molecular and Cell Biology Research Lab, the Shanghai Key Laboratory of Medical Epigenetics, Institutes of Biomedical Sciences, Fudan University, Shanghai, 200032, China

[7]Department of Pharmacology and Moores Cancer Center, University of California San Diego, La Jolla, California, USA

# These authors contributed equally to this work.

* Corresponding author: xuyh@fudan.edu.cn

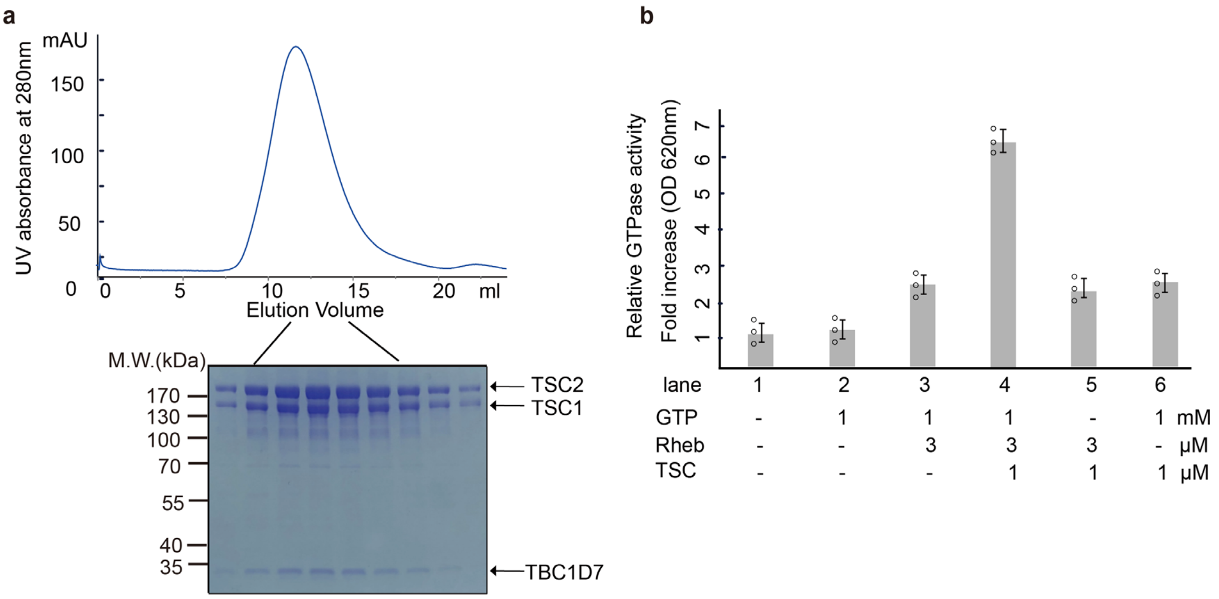

Supplementary Figure 1. TSC complex purification and in vitro GAP activity.

(a) Gel filtration profile of the purified TSC complex on Superose 6 column. The peak fractions were subjected to SDS-PAGE followed by Coomassie blue staining. **(b)** *In vitro* GAP activity assay of TSC complex with purified Rheb as substrate. Malachite green/acid molybdate reagent was used to measure formation of inorganic phosphate. The concentrations of GTP and proteins are indicated. The experiments were performed in triplicates. Vertical axis indicates the fold increase of inorganic phosphate measured by OD 620nm. Data are presented as mean values +/- SEM. The differences between means with $p < 0.01$.

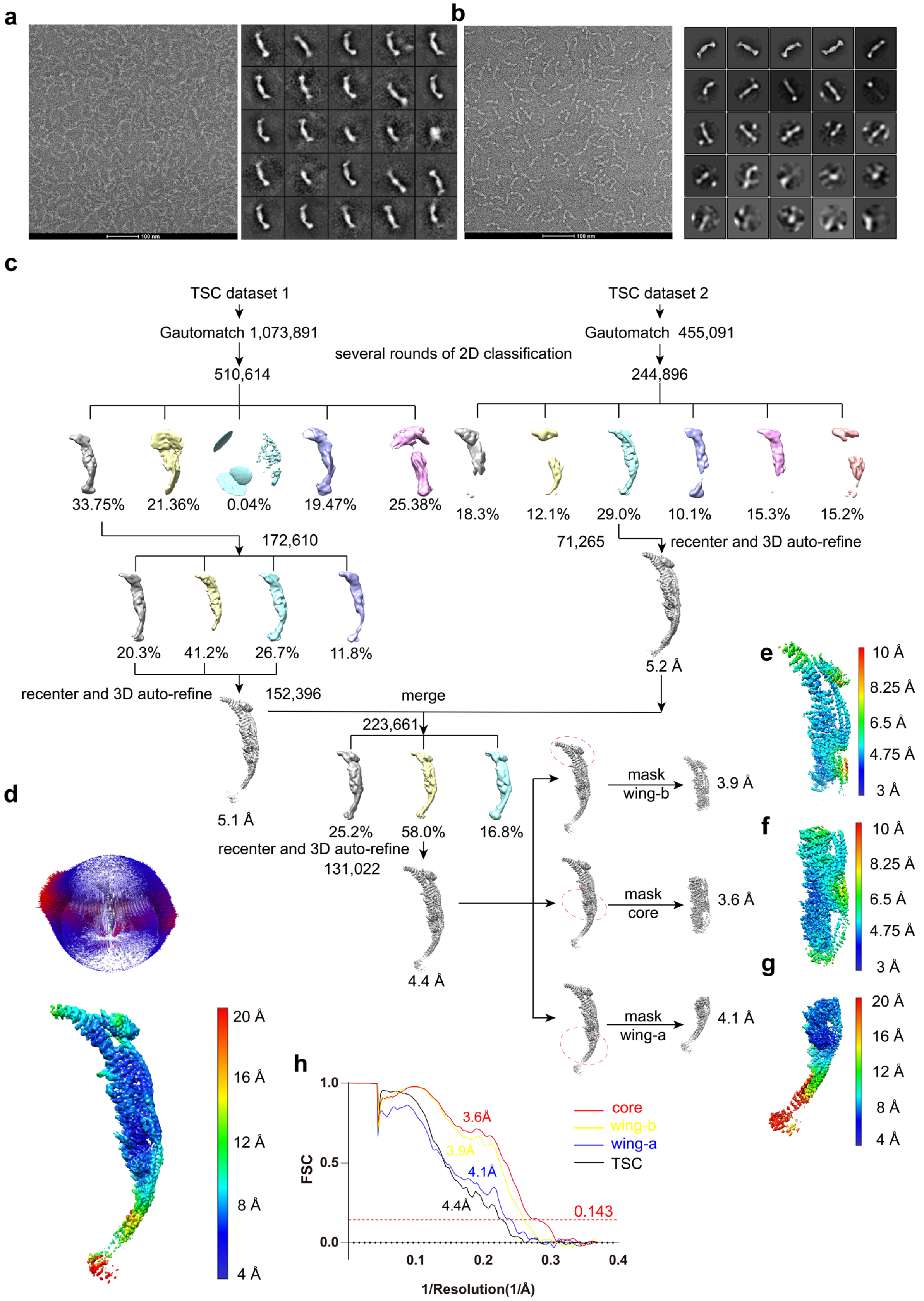

Supplementary Figure 2. Data collection and processing of TSC complex.

(a) Representative negative staining images (left) and 2D classification from 5 times repeatedly TSC complex. (**b)** Representative negative staining images (left) and 2D classification of TSC complex with GraFix from 5 times repeatedly TSC complex. (**c)** Flow-chart of the cryo-EM data processing and 3D reconstructions. (**d)** Local resolution estimation and orientation of the cryo-EM reconstructions of overall TSC complex at 4.4 Å resolution. Local resolution estimations of the wing-b module at 3.9 Å resolution (**e**), of the core module at 3.6 Å resolution (**f**), and of the wing-a module of TSC complex at 4.1 Å resolution (**g**), respectively. (**h)** FSC curves of the best resolved classes of the TSC complex (black), wing-a (blue), core (red) and wing-b (yellow) modules.

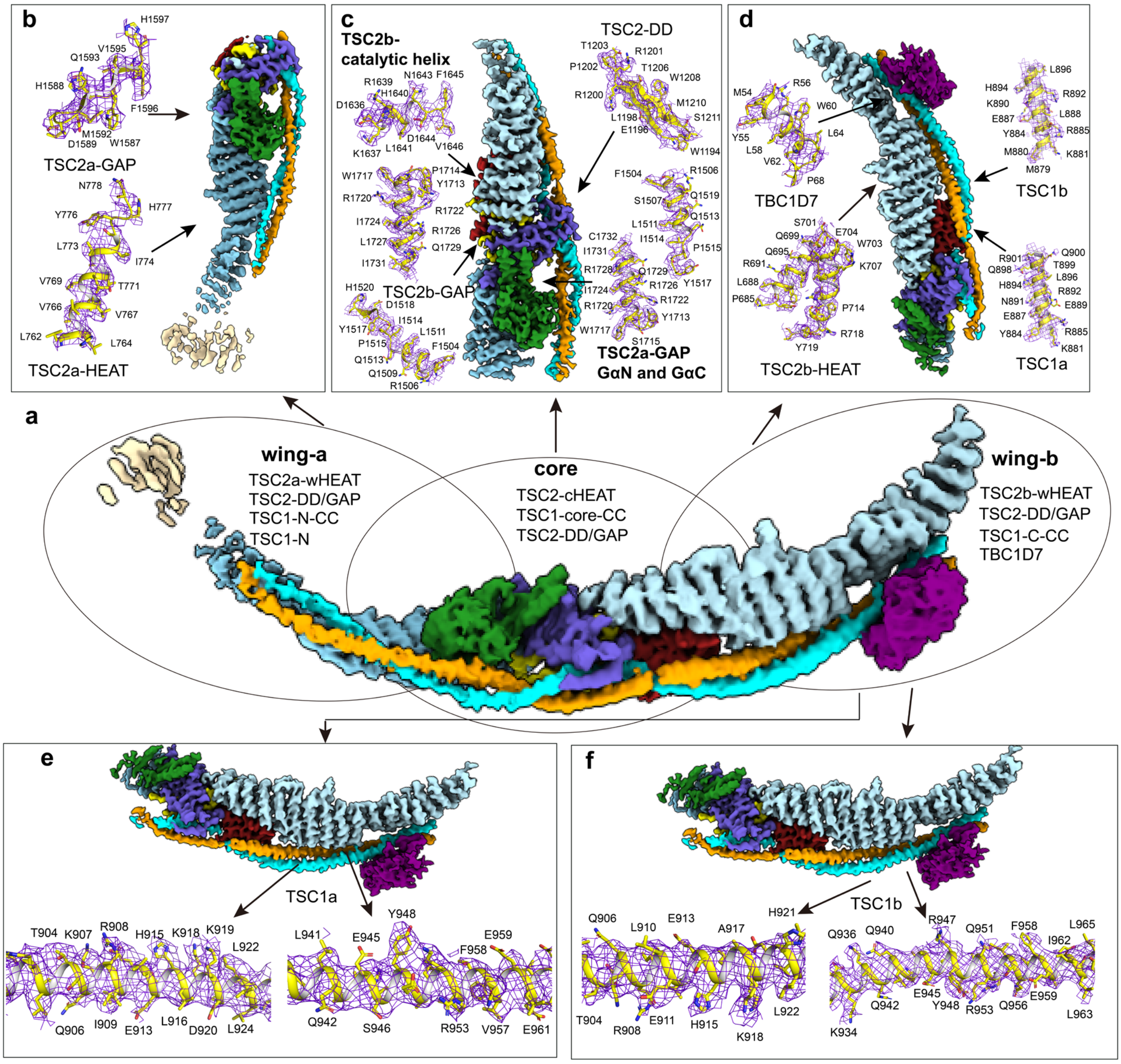

Supplementary Figure 3 Cryo-EM maps and structural model of the TSC complex.

(a) The cryo-EM map of overall TSC complex structure at 4.4 Å resolution with the map colored as in Fig.1b. **b-d,** Locally refined cryo-EM reconstructions of wing-a module at 4.1 Å resolution (**b**), core module at 3.6 Å resolution, in which TSC2 catalytic helix and GαN, GαC helix pair are shown (**c**), and wing-b module at 3.9 Å resolution (**d**). TSC1a C-terminal CC domain (**e**) and TSC1b C-terminal CC domain (**f**) at 3.5-4 Å resolution. Close-up views of the model building are shown, in which the maps are shown in blue meshes, the models are shown in sticks, and residues are labeled.

a

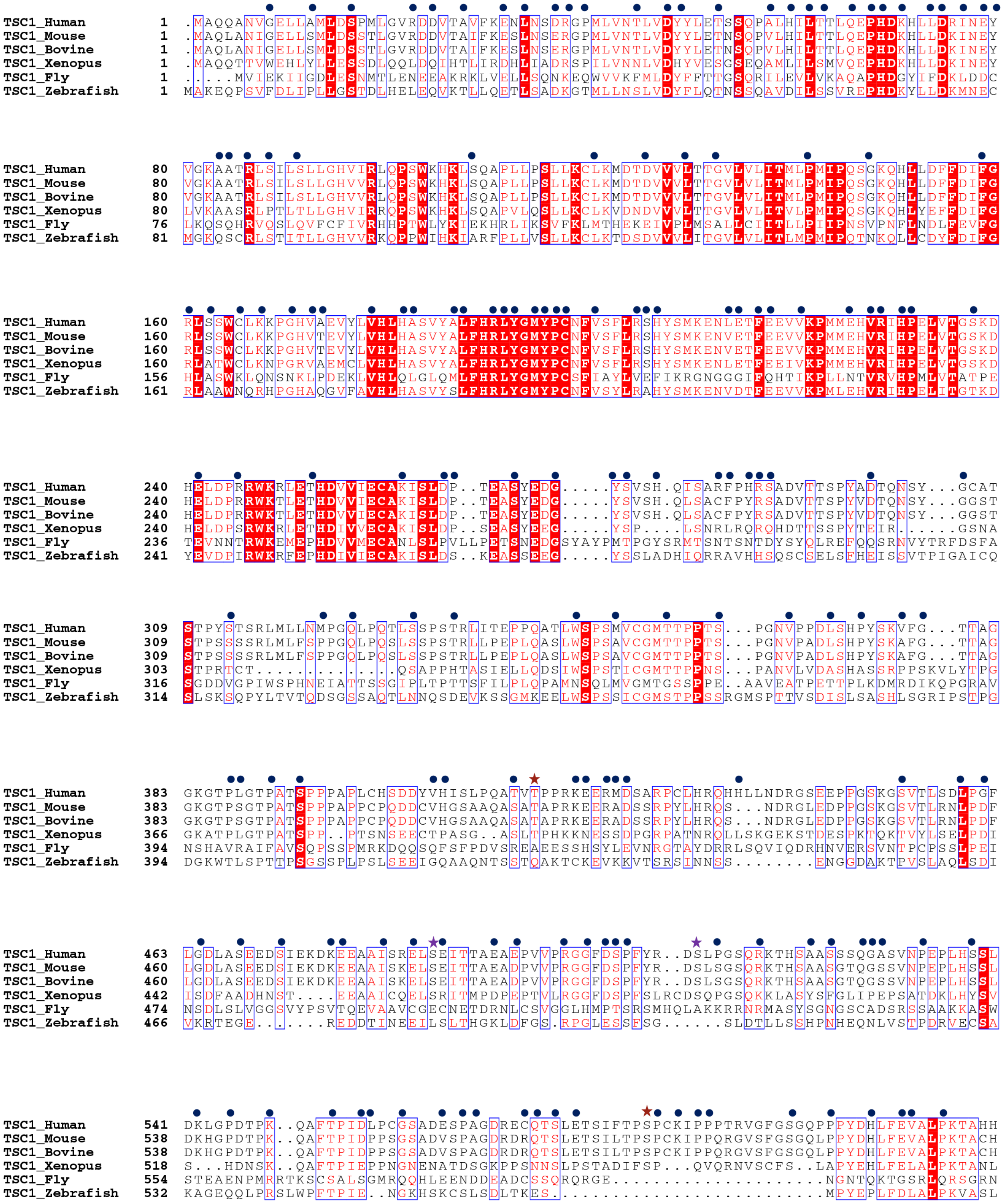

TSC1_Human
TSC1_Mouse
TSC1_Bovine
TSC1_Xenopus
TSC1_Fly
TSC1_Zebrafish

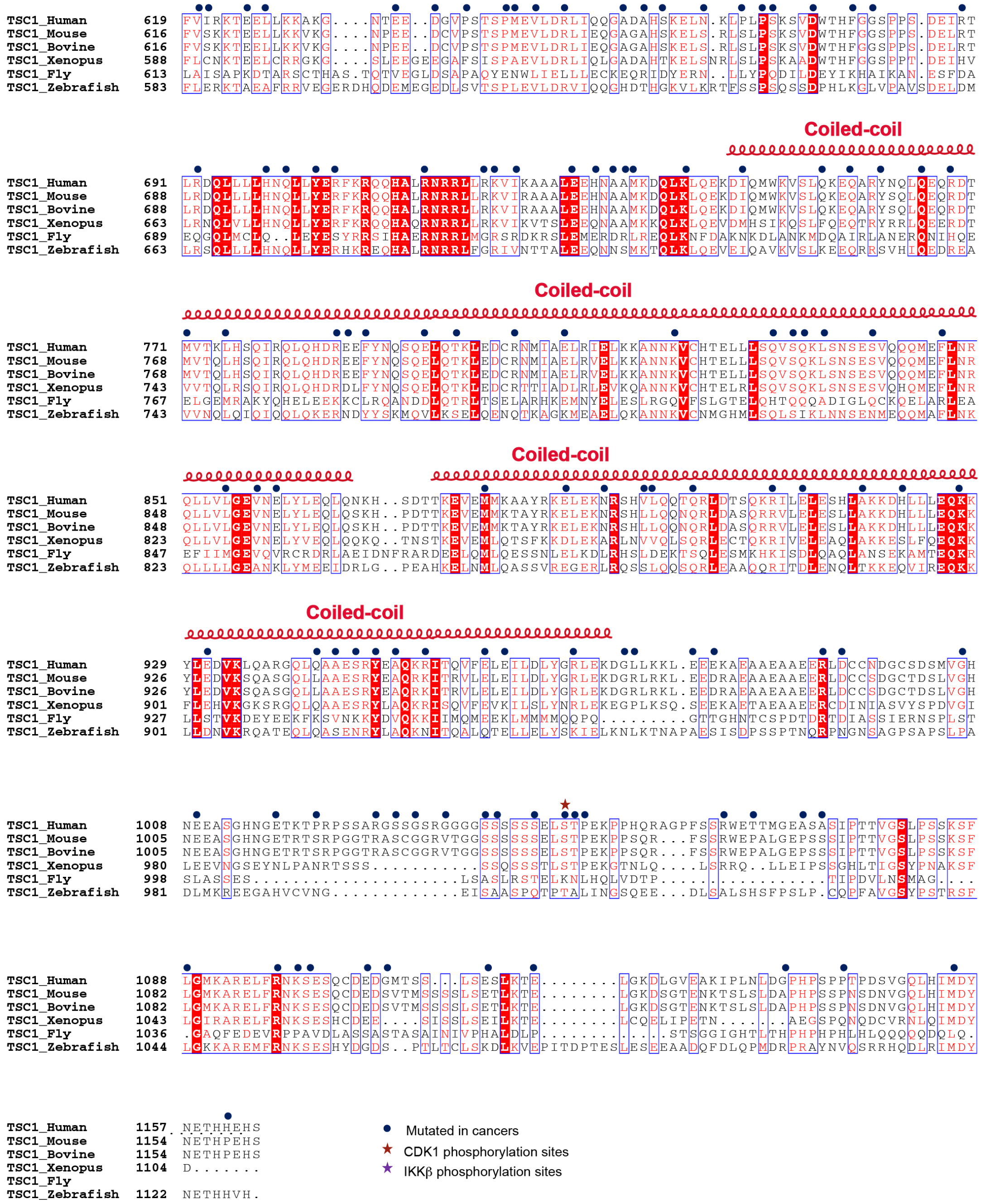

TSC1_Human
TSC1_Mouse
TSC1_Bovine
TSC1_Xenopus
TSC1_Fly
TSC1_Zebrafish
Coiled-coil
Coiled-coil
Coiled-coil
Coiled-coil
Mutated in cancers
CDK1 phosphorylation sites
IKKβ phosphorylation sites

b

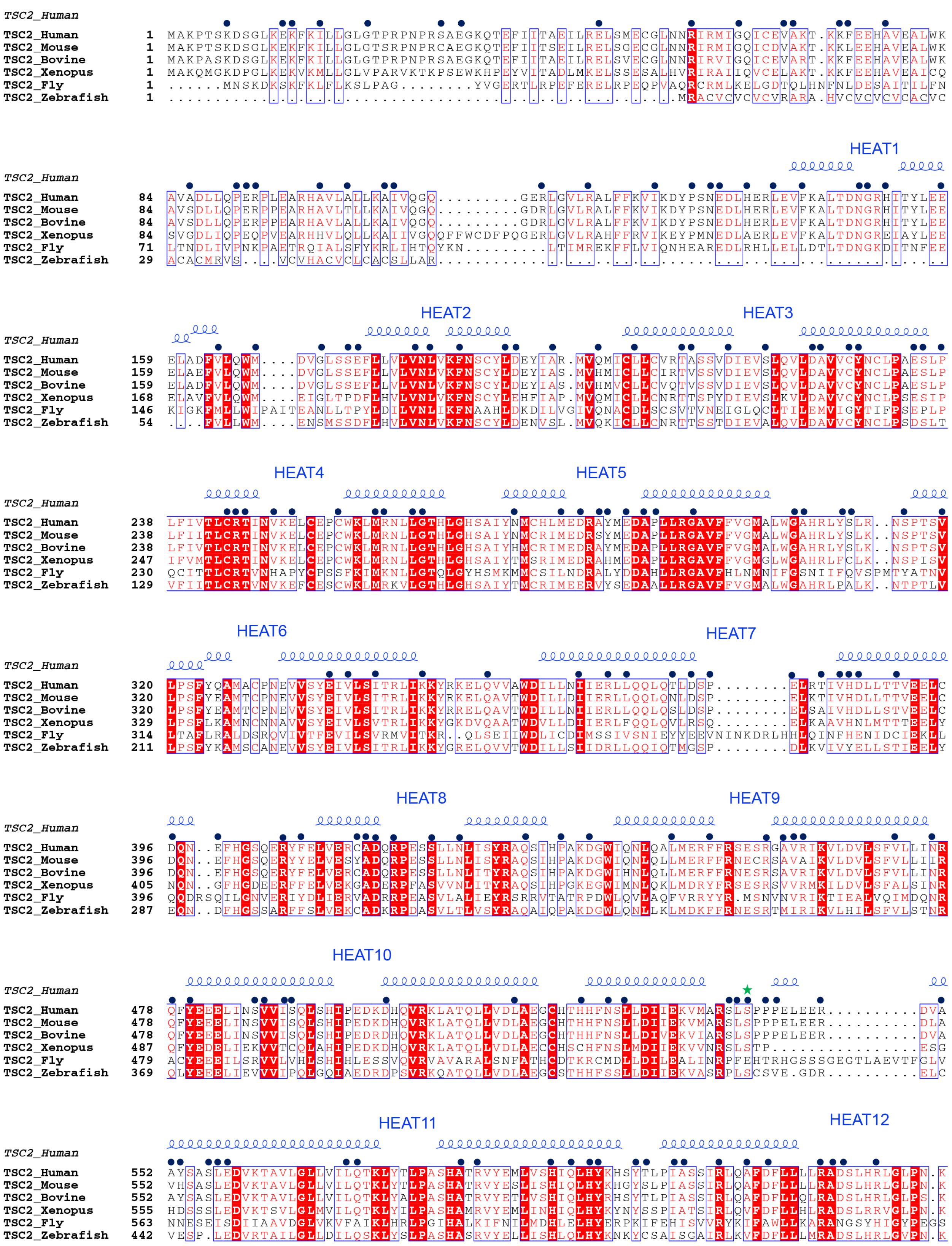
TSC2_Human
TSC2_Mouse
TSC2_Bovine
TSC2_Xenopus
TSC2_Fly
TSC2_Zebrafish
HEAT1
HEAT2
HEAT3
HEAT4
HEAT5
HEAT6
HEAT7
HEAT8
HEAT9
HEAT10
HEAT11
HEAT12

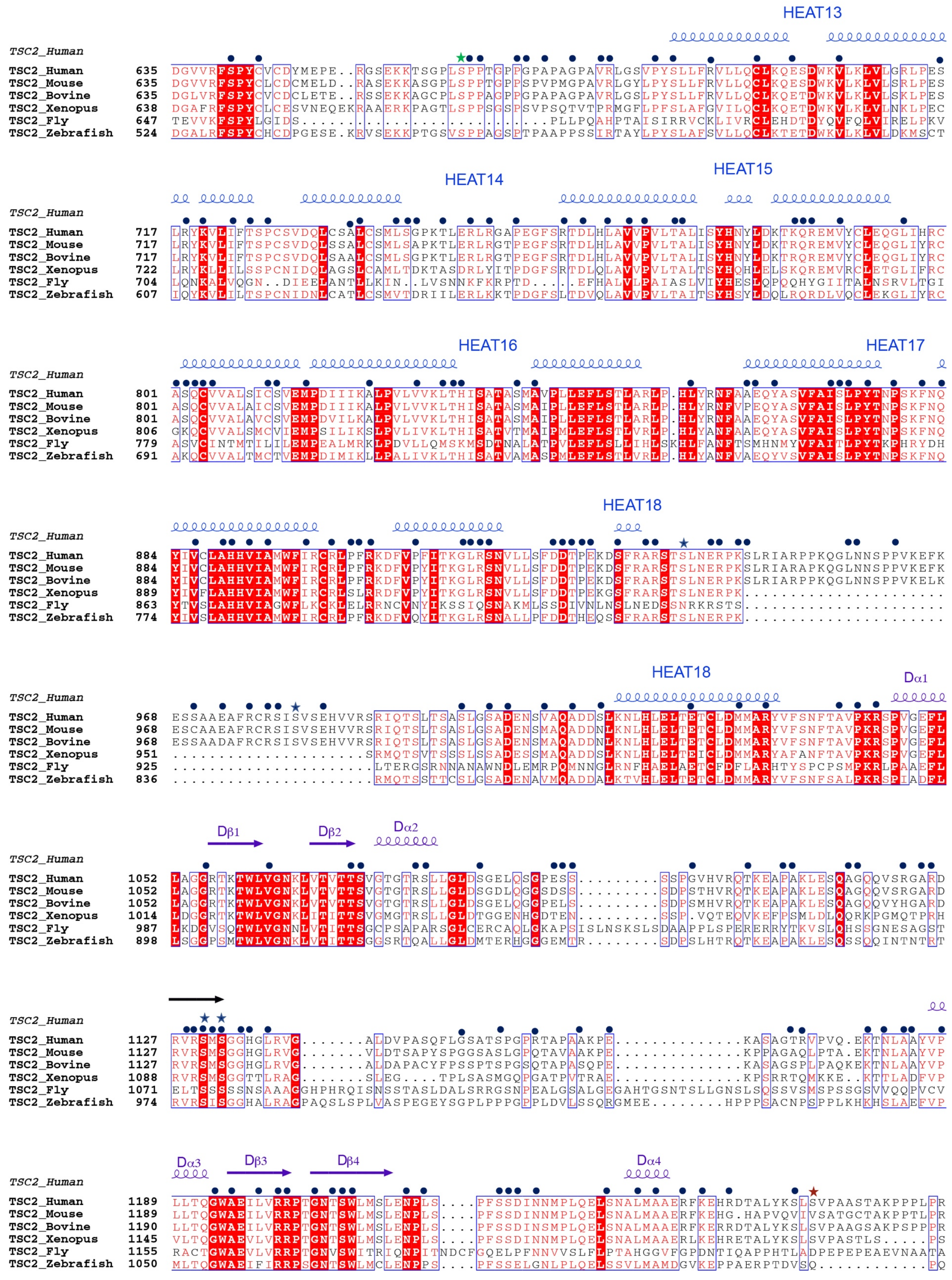
HEAT13
HEAT14
HEAT15
HEAT16
HEAT17
HEAT18
HEAT18
Dα1
Dβ1
Dβ2
Dα2
Dα3
Dβ3
Dβ4
Dα4
TSC2_Human
TSC2_Mouse
TSC2_Bovine
TSC2_Xenopus
TSC2_Fly
TSC2_Zebrafish

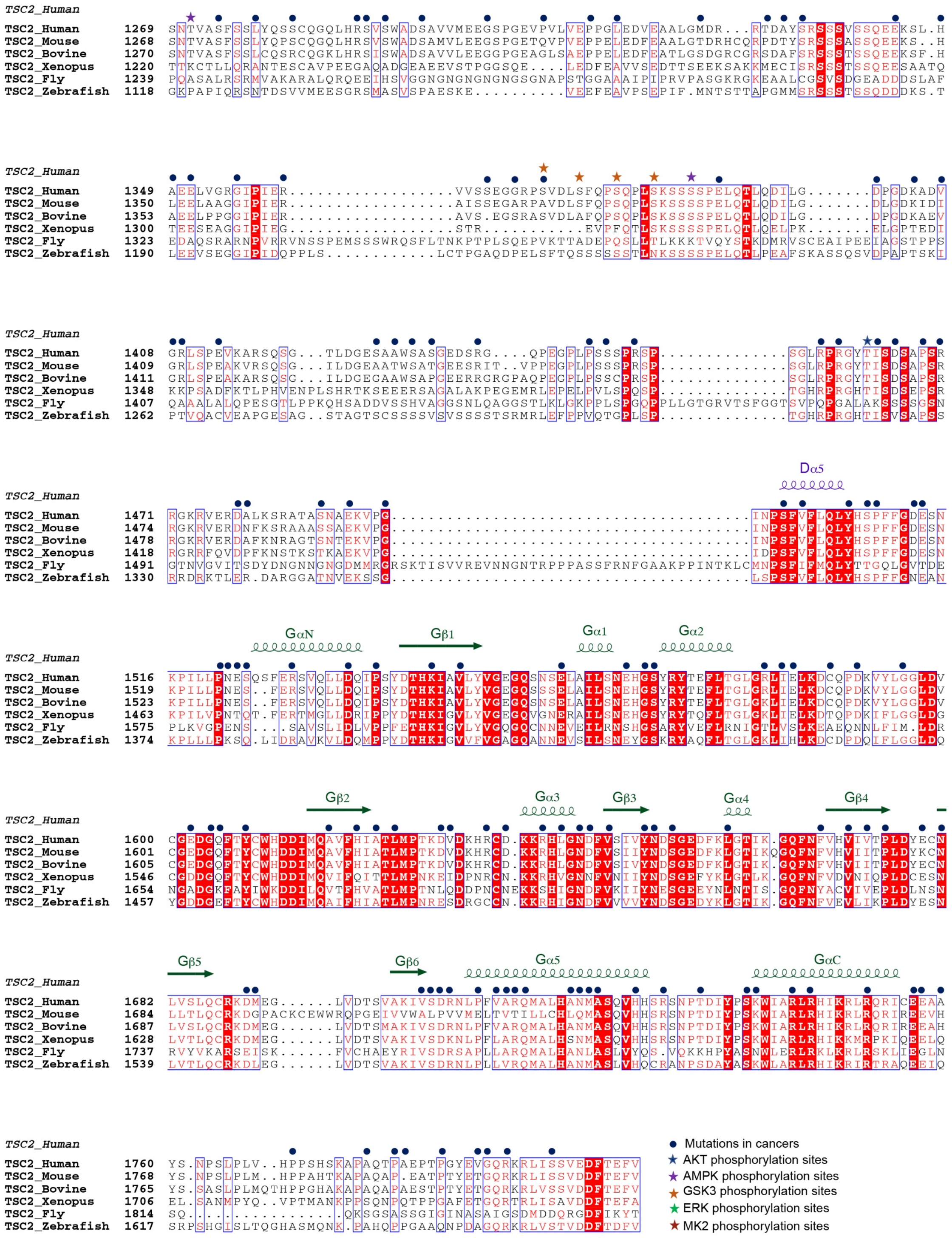
TSC2_Human
TSC2_Mouse
TSC2_Bovine
TSC2_Xenopus
TSC2_Fly
TSC2_Zebrafish
Dα5
GαN
Gβ1
Gα1
Gα2
Gβ2
Gα3
Gβ3
Gα4
Gβ4
Gβ5
Gβ6
Gα5
GαC
Mutations in cancers
AKT phosphorylation sites
AMPK phosphorylation sites
GSK3 phosphorylation sites
ERK phosphorylation sites
MK2 phosphorylation sites

c

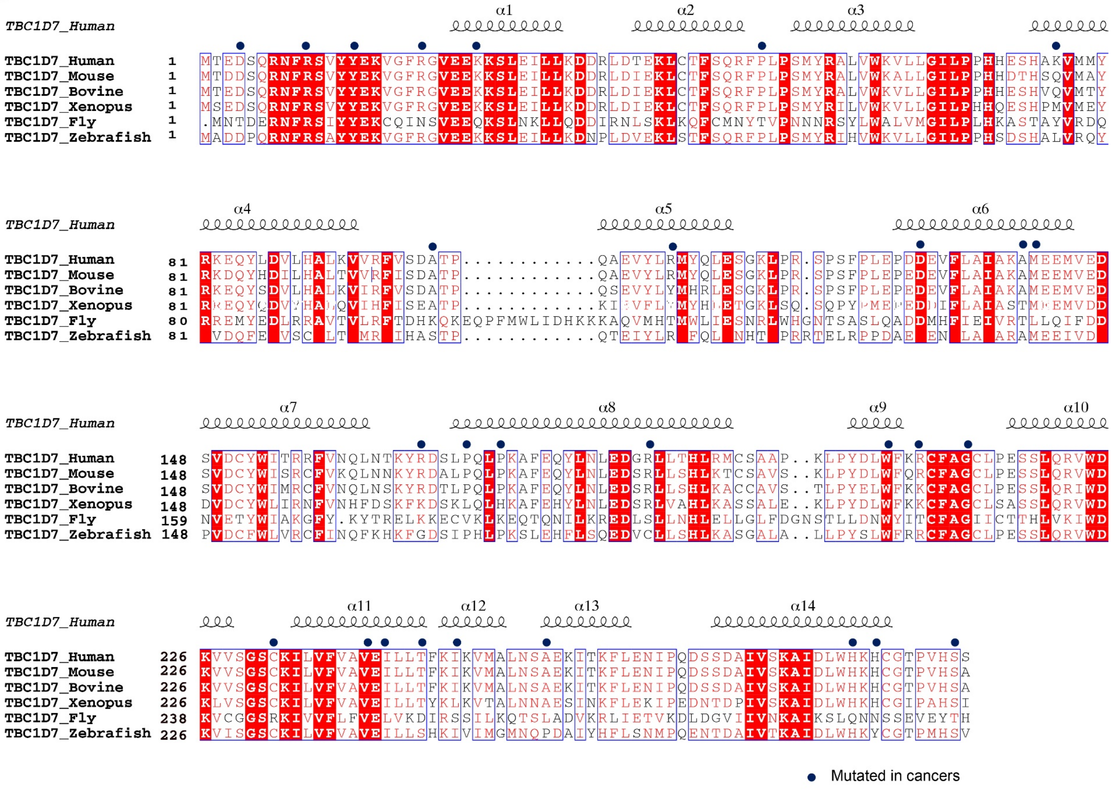

Supplementary Figure 4. Sequence alignment of TSC1, TSC2 and TBC1D7 across species.

Structure-based sequence alignment of TSC1 (**a**), TSC2 (**b**), and TBC1D7 (**c**) across species. Secondary structure (α helices and β strands) and domain annotations are indicated above the sequences. Phosphorylation sites and disease-associated mutations from COSMIC database[1] are marked in stars and circles, respectively. Alignments were generated by ESPript[2].

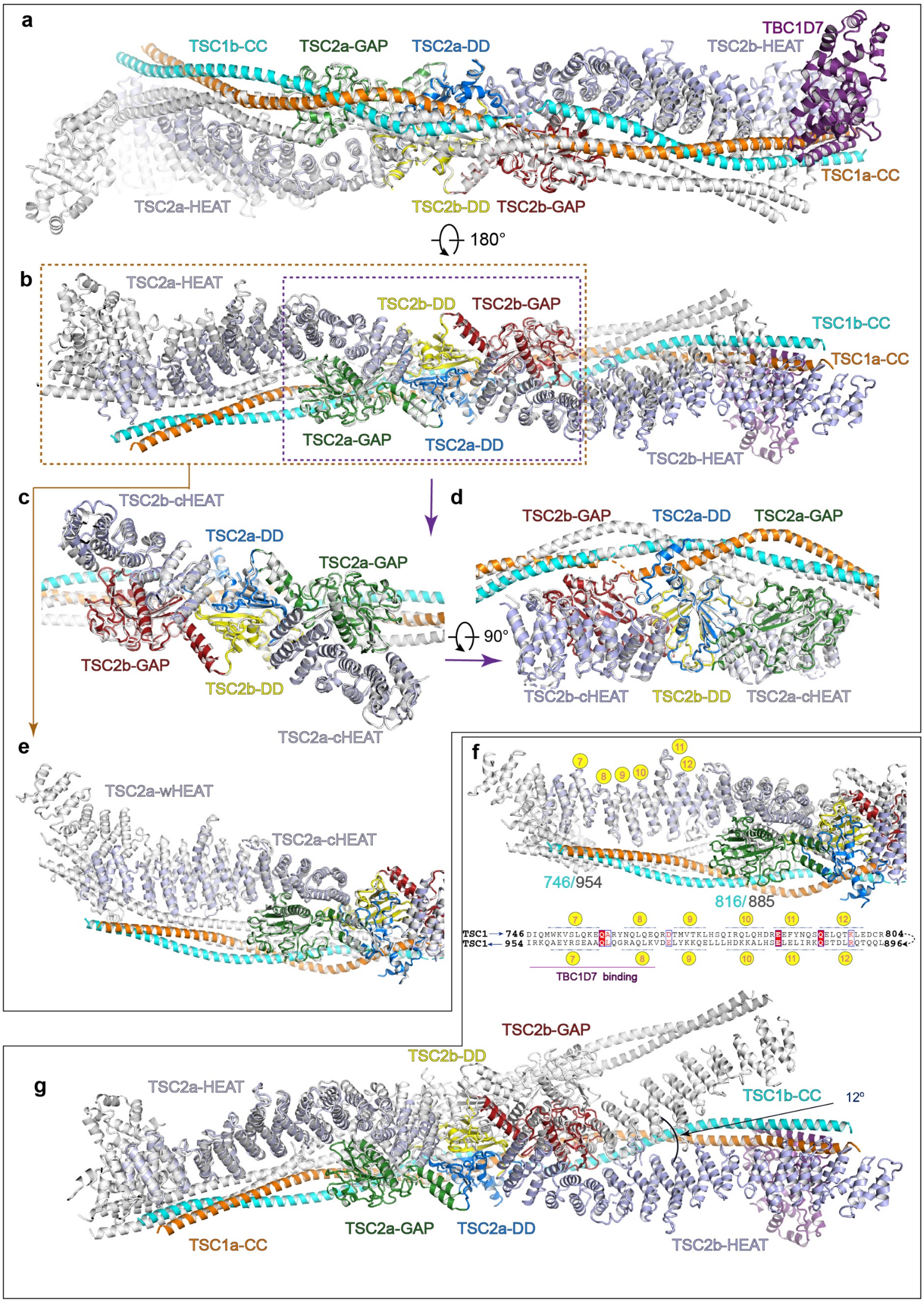

Supplementary Figure 5. Asymmetric assembly of TSC complex.

(a-e) Reciprocal superimposition of two TSC complexes with one complex colored as in Fig. 1 and the other in grey (**a-b**). The central core module adopts almost symmetric fold (**c-d**) whereas the wing modules are asymmetrically arranged (**e**). (**f-g**) When two wHEAT modules are superimposed

(**f**), the two TSC complexes show a ~12-degree deviation at the other end (**g**). The asymmetric assembly of TSC complex is resulted from directional binding of TSC1 dimer, as indicated by the schematic figure of TSC1-TSC2 and TSC1-TBC1D7 interactions (**f**).

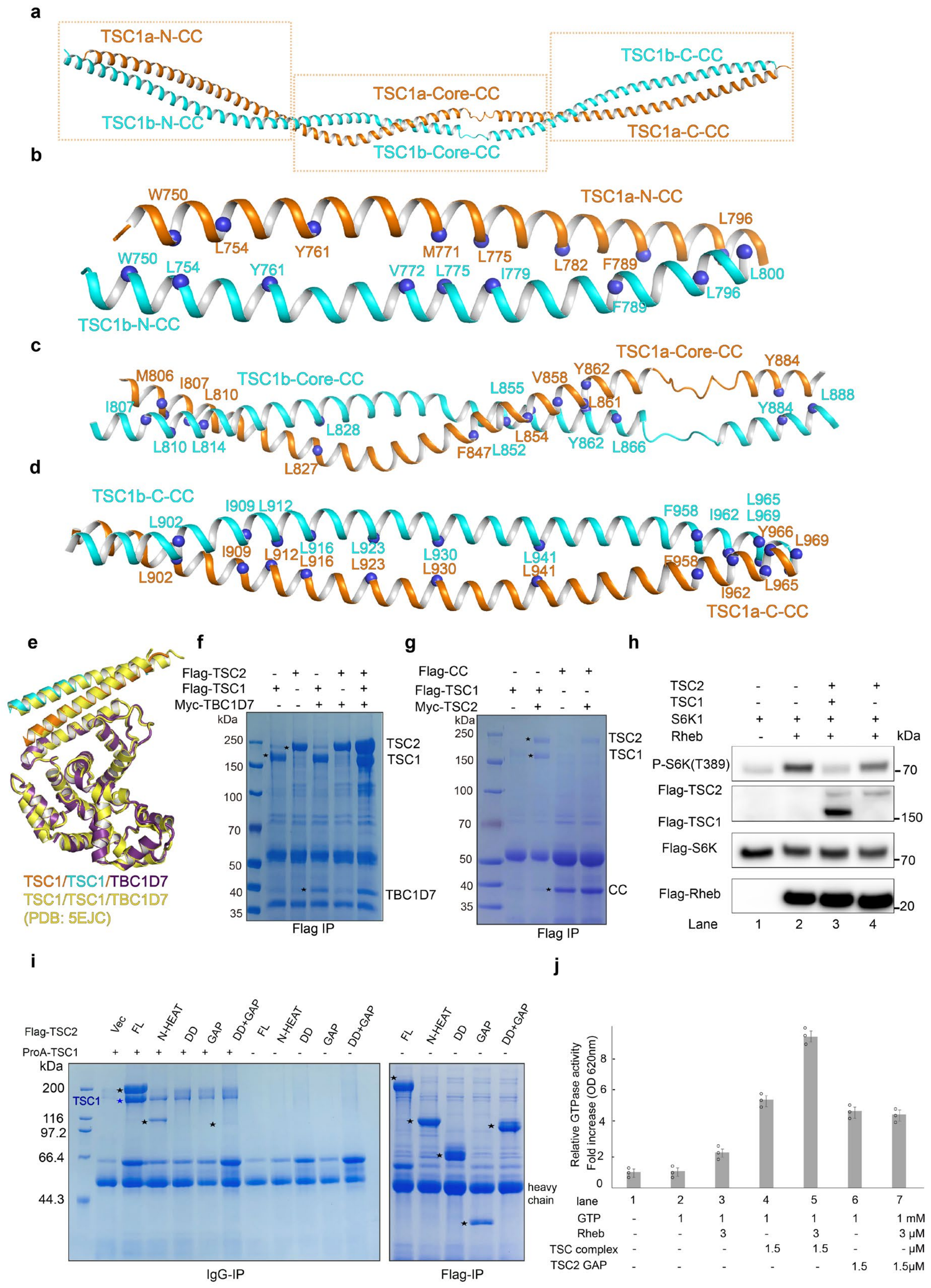

Supplementary Figure 6. TSC1 dimer interface and TSC1 CC binding to TSC2.

(a) The TSC1 coiled-coil dimer is shown, the view is different from Fig. 1c. N-CC, Core-CC and C-CC three parts are labeled. The close-up views of the three interfaces around the N-terminal (**b**), central (**c**), and C-terminal (**d**) regions. The positions of residues involved in dimerization are indicated with blue balls. (**e)** Superimposition of TSC1-TBC1D7 subcomplex in our structure and the previously reported crystal structure (colored in yellow) (PDB:5EJC). **(f)** Flag immunoprecipitation assay. Flag-TSC1, Flag-TSC2, Myc-TBC1D7 were transfected alone or co-transfected to Expi293F cells and purified on anti-Flag M2-beads. The bound proteins were subjected to SDS-PAGE followed by Coomassie blue staining. **(g)** Flag immunoprecipitation assay performed as in (f). Flag-TSC1 or Flag-TSC1 CC (725-988aa) were co-transfected with myc-TSC2 to Expi293F cells and purified on anti-Flag M2-beads. The bound proteins were subjected to SDS-PAGE followed by Coomassie blue staining. (**h)** Cell-base GAP activity assay of TSC2 with or without TSC1. The activities were detected by western blotting with antibody against phosphorylated S6K (T389). Source data are provided as a Source Data file for uncropped blots. **(i)** Flag-tagged TSC2 full-length, N-HEAT, DD, GAP and DD-GAP domains of TSC2 were transfected or co-transfected with Protein A-tagged TSC1 to Expi293F cells and purified on anti-IgG beads. The Flag-IP indicates the position of each TSC2 construct. The bound proteins were subjected to SDS-PAGE followed by Coomassie blue staining. **(j)** In vitro GAP activity of TSC complex and TSC2 GAP domain. The experiments were performed in triplicates. Vertical axis indicates the fold increase of inorganic phosphate measured by OD 620nm. Data are presented as mean values +/ SEM. The differences between means with $p < 0.01$.

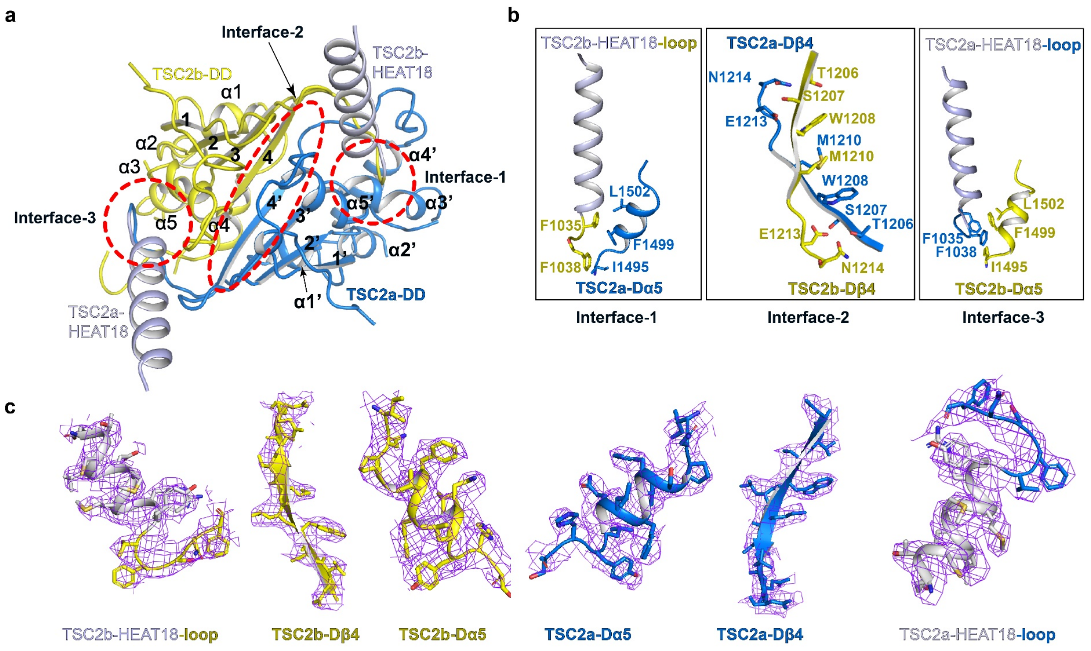

Supplementary Figure 7. Three interfaces of TSC2 dimer.

Cartoon model of TSC2 dimerization domain (**a**) and close-up views of the three interfaces (**b**). Secondary structural elements are labeled with numbers with apostrophe in the TSC2a. Critical residues involved in dimerization are shown in sticks (b) and the corresponding cryo-EM maps are shown in blue meshes (**c**).

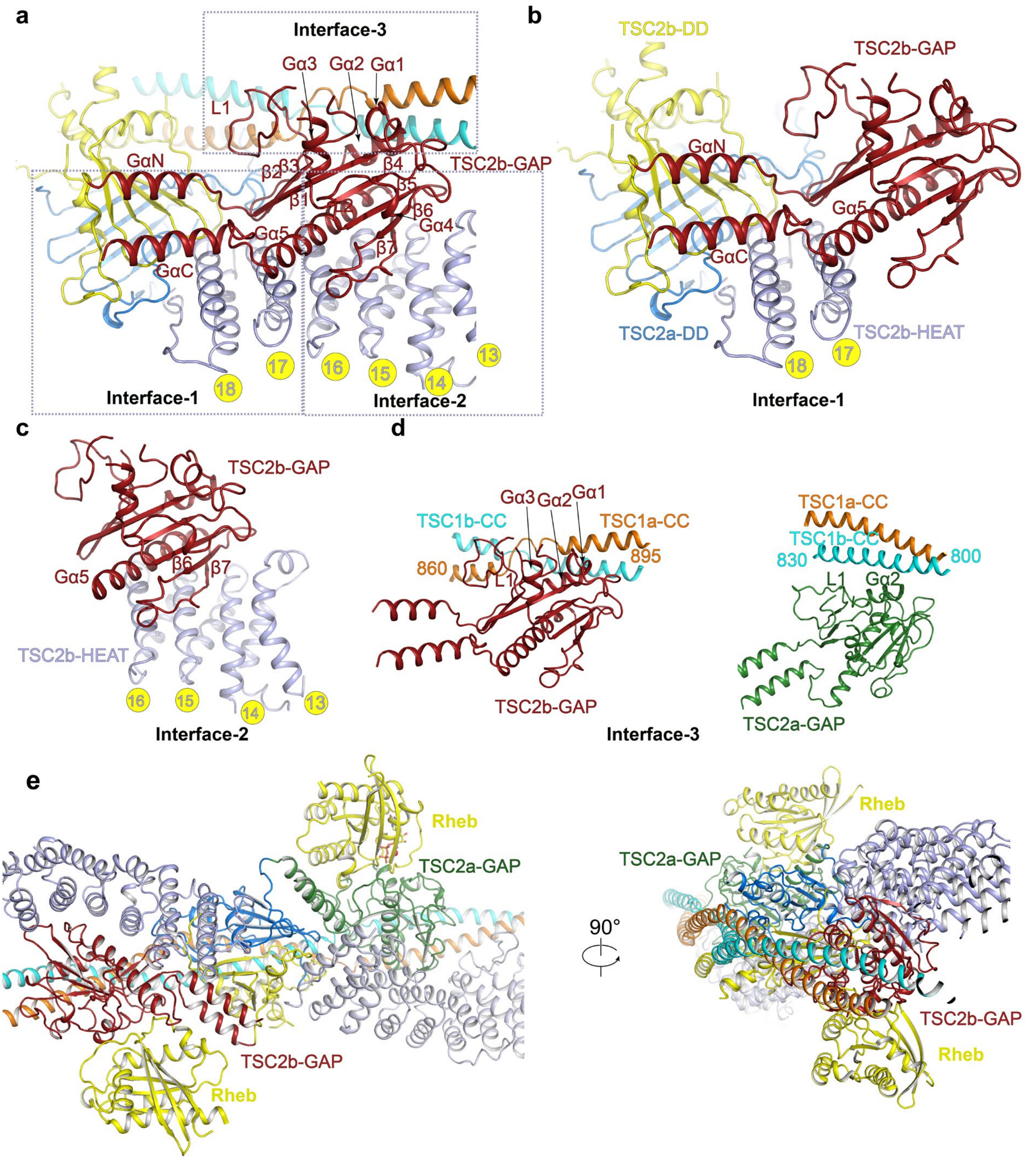

Supplementary Figure 8. Detailed interactions of TSC2 GAP domain within other modular of TSC complex.

(a) Interactions between TSC2 GAP domain and the rest of TSC complex. The interfaces shown in b-d are highlighted with dashed boxes. **(b-d)** Close-up views of the three interfaces as indicated in (a). Note that interface-1 and interface-2 are the same for two GAP domains, whereas interface-

3 (d) is different in TSC2a and TSC2b. (**e)** Putative interactions between TSC2 and Rheb based on alignment with Rap1-Rap1GAP structure in two views.

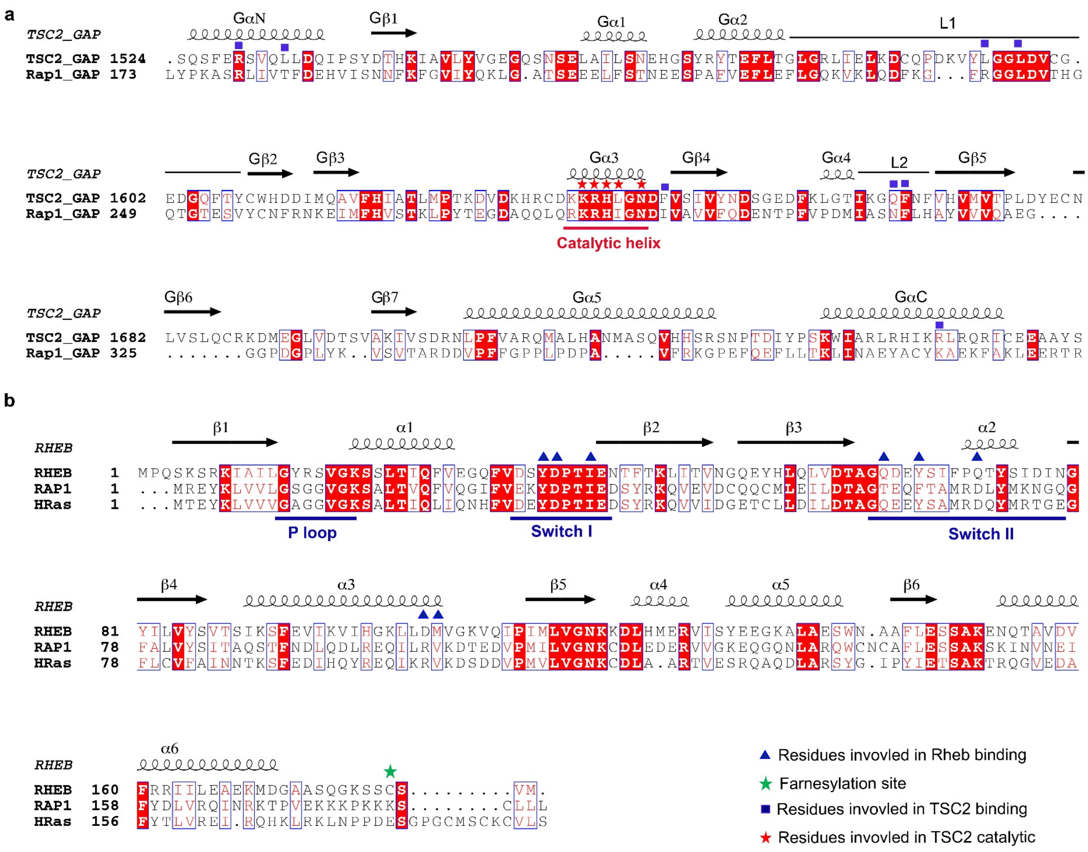

Supplementary Figure 9. Sequence alignments of TSC2 GAP domain and Rheb.

(a) Structure-based sequence alignment of human TSC2 GAP and Rap1 GAP. Secondary structures are indicated above the sequences. Residues involved in TSC2 GAP catalytic and binding of Rheb are indicated with red stars and blue squares, respectively. (**b)** Sequence alignment of human Rheb, Rap1 and H-Ras. The critical elements of GTPase including the P-loop, switch I, and switch II regions are indicated. Residue annotations are indicated below.

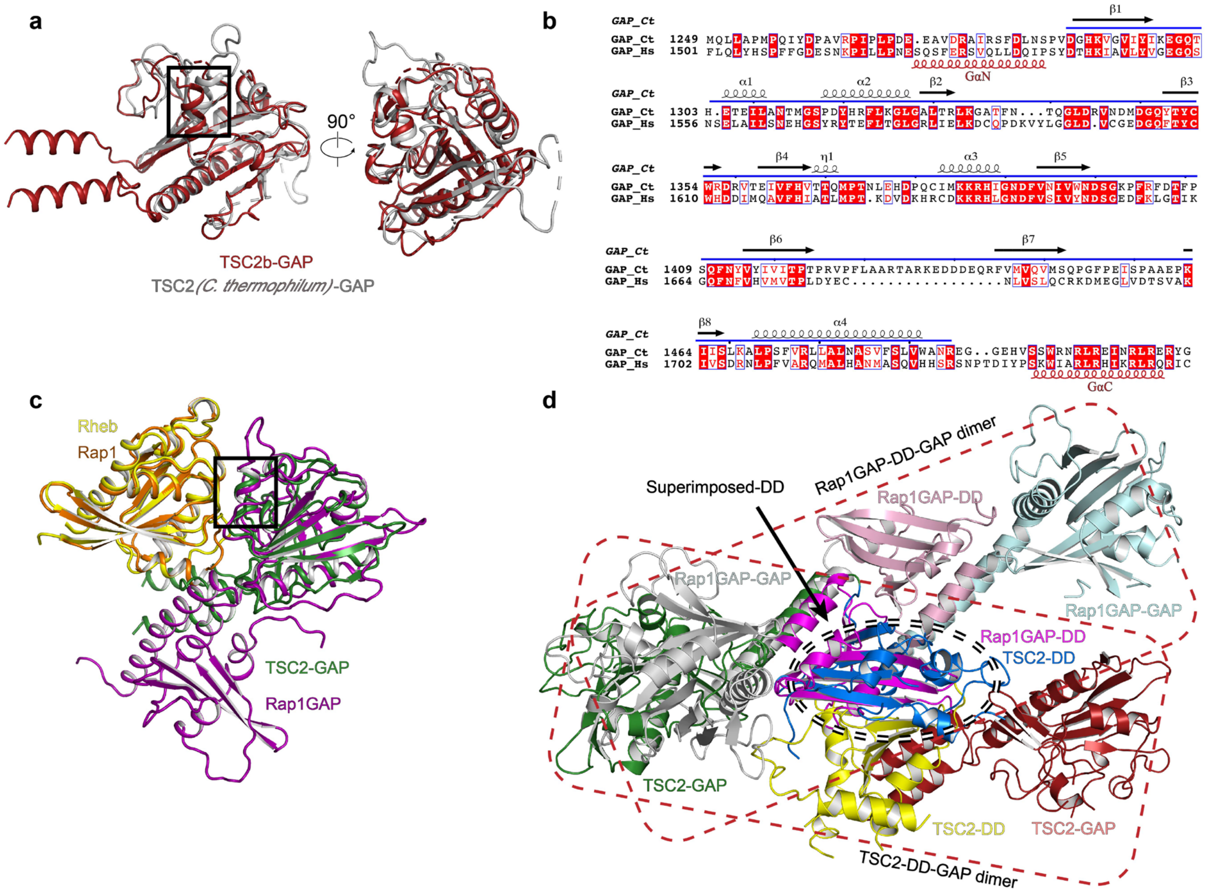

Supplementary Figure 10. Structural comparison of GAP and DD domains of TSC2 and related proteins.

(a) Structural comparison of human TSC2 GAP (red) and *C. thermophilum* GAP (grey). The catalytic helices are in different conformation and are highlighted with an empty box. **(b)** Sequence alignment of human (*homo sapiens*, Hs) TSC2 GAP and *C. thermophilum* (Ct) TSC2 GAP. Secondary structures are indicated above the sequences. Note that the sequence of *C. thermophilum* TSC2 construct used for structure determination is indicated in blue line, which doesn't contain GαN and GαC (resolved from our TSC2 structure, red label below). **(c)** Structural comparison of human TSC2 GAP/Rheb and Rap1GAP/Rap1. The catalytic helices are in a similar conformation and highlighted with an empty box. **(d)** Structural comparison of TSC2 DD-GAP dimer with Rap1GAP dimer based on alignment of one DD. Each domain is shown in indicated color.

Supplementary References:

1 Tate, J. G. *et al.* COSMIC: the Catalogue Of Somatic Mutations In Cancer. *Nucleic Acids Res* **47**, D941-D947, doi:10.1093/nar/gky1015 (2019).

2 Robert, X. & Gouet, P. Deciphering key features in protein structures with the new ENDscript server. *Nucleic Acids Res* **42**, W320-324, doi:10.1093/nar/gku316 (2014).
