# Supplementary material for: Structural insights into TSC complex assembly and GAP activity on Rheb
Source: Nat Commun. 2021 Jan 12;12:339. doi: 10.1038/s41467-020-20522-4 (PMC7804450; doi:10.1038/s41467-020-20522-4)
Supplement: Supplementary file 6 — Reporting Summary [file 41467_2020_20522_MOESM6_ESM.pdf]

Corresponding author(s): Yanhui Xu

Last updated by author(s): Nov 23, 2020

Reporting Summary

Nature Research wishes to improve the reproducibility of the work that we publish. This form provides structure for consistency and transparency in reporting. For further information on Nature Research policies, see our Editorial Policies and the Editorial Policy Checklist.

Statistics

For all statistical analyses, confirm that the following items are present in the figure legend, table legend, main text, or Methods section.

n/a	Confirmed	
☐	☒	The exact sample size (n) for each experimental group/condition, given as a discrete number and unit of measurement
☐	☒	A statement on whether measurements were taken from distinct samples or whether the same sample was measured repeatedly
☒	☐	The statistical test(s) used AND whether they are one- or two-sided<br>*Only common tests should be described solely by name; describe more complex techniques in the Methods section.*
☒	☐	A description of all covariates tested
☒	☐	A description of any assumptions or corrections, such as tests of normality and adjustment for multiple comparisons
☒	☐	A full description of the statistical parameters including central tendency (e.g. means) or other basic estimates (e.g. regression coefficient) AND variation (e.g. standard deviation) or associated estimates of uncertainty (e.g. confidence intervals)
☒	☐	For null hypothesis testing, the test statistic (e.g. F, t, r) with confidence intervals, effect sizes, degrees of freedom and P value noted<br>*Give P values as exact values whenever suitable.*
☒	☐	For Bayesian analysis, information on the choice of priors and Markov chain Monte Carlo settings
☒	☐	For hierarchical and complex designs, identification of the appropriate level for tests and full reporting of outcomes
☒	☐	Estimates of effect sizes (e.g. Cohen's d, Pearson's r), indicating how they were calculated

Our web collection on statistics for biologists contains articles on many of the points above.

Software and code

Policy information about availability of computer code

Data collection	The cryo-EM grids of TSC were loaded onto a Thermo Fisher Scientific Titan Krios transmission electron microscope equipped with a Gatan GIF Quantum energy filter (slit width 20eV) and operating at 300 kV for data collection. All the cryo-EM images were automatically recorded by a post-GIF Gatan K2 Summit direct electron detector in the super-resolution counting mode using Serial-EM49 with a nominal magnification of 105,000 x in the EFTEM mode, which yielded a super-resolution pixel size of 0.678 Å on the image plane, and with a defocus ranged from 1.0 to 3.5 µm. Each micrograph stack was dose-fractionated to 32 frames with a total electron dose of ~ 50 e-/ Å2 and a total exposure time of 11.49 s. For the first dataset of TSC sample, 3,316 micrographs from a total of 3,605 micrographs were selected for further processing. As for the second dataset of TSC sample, 1,381 micrographs from a total of 1,546 micrographs were selected for further processing.
Data analysis	For cryo-EM data, drift and beam-induced motion correction were applied on the super-resolution movie stacks using MotionCor2 and binned two fold to a calibrated pixel size of 1.356 Å/pix. The defocus values were estimated by Gctf from summed images without dose weighting. Other procedures of cryo-EM data processing were performed within RELION v3.0 using the dose-weighted micrographs.

For manuscripts utilizing custom algorithms or software that are central to the research but not yet described in published literature, software must be made available to editors and reviewers. We strongly encourage code deposition in a community repository (e.g. GitHub). See the Nature Research guidelines for submitting code & software for further information.

Data

Policy information about availability of data

All manuscripts must include a data availability statement. This statement should provide the following information, where applicable:

- Accession codes, unique identifiers, or web links for publicly available datasets
- A list of figures that have associated raw data
- A description of any restrictions on data availability

Cryo-EM maps and atomic coordinates have been deposited and the coordinate numbers will be updated soon.

Field-specific reporting

Please select the one below that is the best fit for your research. If you are not sure, read the appropriate sections before making your selection.

☒ Life sciences ☐ Behavioural & social sciences ☐ Ecological, evolutionary & environmental sciences

For a reference copy of the document with all sections, see nature.com/documents/nr-reporting-summary-flat.pdf

Life sciences study design

All studies must disclose on these points even when the disclosure is negative.

Sample size	No sample size was calculated in our manuscript.
Data exclusions	No data exclusions were involved in our manuscript.
Replication	The in vitro GAP experiments for figure S1b and S6j were performed in triplicates.
Randomization	No randomization was involved in our manuscript.
Blinding	No blinding was involved in our manuscript.

Reporting for specific materials, systems and methods

We require information from authors about some types of materials, experimental systems and methods used in many studies. Here, indicate whether each material, system or method listed is relevant to your study. If you are not sure if a list item applies to your research, read the appropriate section before selecting a response.

Materials & experimental systems

n/a	Involved in the study	
☐	☒	Antibodies
☐	☒	Eukaryotic cell lines
☒	☐	Palaeontology and archaeology
☒	☐	Animals and other organisms
☒	☐	Human research participants
☒	☐	Clinical data
☒	☐	Dual use research of concern

Methods

n/a	Involved in the study	
☒	☐	ChIP-seq
☒	☐	Flow cytometry
☒	☐	MRI-based neuroimaging

Antibodies

Antibodies used	phosphorylated-S6K (Thr 389) antibody, flag-HRP antibody (Sigma, A8592), goat anti-rabbit IgG-HRP (Abmart, M21002L)
Validation	1. phosphorylated-S6K (Thr 389) antibody (rabbit, CST #9205), https://www.cellsignal.com/products/primary-antibodies/phospho-p70-s6-kinase-thr389-antibody/9205<br>2. flag-HRP antibody, https://www.sigmaaldrich.com/catalog/product/sigma/a8592

Eukaryotic cell lines

Policy information about cell lines

Cell line source(s)	Expi293F cells and HEK293A cells

Authentication	We used commercial Expi293F cell line from ThermoFisher (A14527).
Mycoplasma contamination	The cell line was not tested for mycoplasma contamination.
Commonly misidentified lines<br>(See ICLAC register)	No misindentified cell lines were used in this study.
