# Supplementary material for: Structural insights into TSC complex assembly and GAP activity on Rheb
Source: Nat Commun. 2021 Jan 12;12:339. doi: 10.1038/s41467-020-20522-4 (PMC7804450; doi:10.1038/s41467-020-20522-4)
Supplement: Supplementary file 7 — Description of Additional Supplementary Files [file 41467_2020_20522_MOESM7_ESM.docx]

Description of Additional Supplementary Files

Title: Supplementary Movie 1

Description : The TSC complex overall map at a resolution of 4.4 Å

Title: Supplementary Movie 2

Description: The TSC complex wing-a map at a resolution of 4.1 Å

Title: Supplementary Movie 3

Description: The TSC complex core map at a resolution of 3.6 Å

Title: Supplementary Movie 4

Description: The TSC complex wing-b map at a resolution of 3.9 Å
